# Profiles of multisource support and teaching efficacy among teachers in Chinese private universities: a latent profile analysis

**DOI:** 10.3389/fpsyg.2026.1961537

**Published:** 2026-09-28

**Authors:** Huimin Feng, Nannan Wang, Xing Ming

**Affiliations:** 1 Faculty of International Education, Qingdao Hengxing University of Science and Technology, Qingdao, Shandong, China; 2 Office of Academic Affairs, Qingdao Hengxing University of Science and Technology, Qingdao, Shandong, China

**Keywords:** China, latent profile analysis, multisource support, person-centered approach, private university teachers, teaching efficacy

## Abstract

Teaching efficacy is an important psychological belief concerning teachers’ judgments of instructional capability and effectiveness. Most existing research on workplace support received by teachers has adopted a variable-centered approach, focusing primarily on average associations between individual sources of support and teacher outcomes, while paying less attention to heterogeneity in how multisource support resources are configured within teachers. Using survey data from 400 teachers at six private universities in Qingdao, China, this study treated emotional and material support from the organization, leaders, and colleagues as profile indicators. Latent Profile Analysis (LPA) was used to identify configurations of teacher support resources, and the BCH method was used to compare General Teaching Efficacy (GTE) and Personal Teaching Efficacy (PTE) across profiles. The selected six-profile solution differentiated six sample-relative patterns: the Very Low Overall Support Profile, Low Overall Support Profile, Moderate Balanced Support Profile, Relational Support-Dominant Profile, Organizational Support-Dominant Profile, and High Balanced Support Profile. The profiles differed significantly in both dimensions of teaching efficacy. The Very Low Overall Support Profile had the lowest means, whereas the Organizational Support-Dominant Profile and High Balanced Support Profile had relatively high means. A series of robustness and sensitivity analyses supported the broader profile patterns, although exact profile boundaries and membership showed some model sensitivity. The findings indicate that support environments in private universities vary in both overall resource levels and asymmetric configurations across support sources. By contrast, independent differentiation between emotional and material support within the same source was less pronounced. The findings suggest that teacher support practices should consider both the adequacy of available resources and how support from different sources is combined, with multidimensional support-needs assessments focusing on patterns of resource adequacy across organizational, leadership, and colleague support rather than on fixed profile classifications.

## 1 Introduction

Teaching efficacy reflects teachers’ cognitive judgments about whether teaching activities can be implemented effectively and produce the intended educational effects. Following the two-factor framework proposed by Gibson and Dembo (1984), this study uses teaching efficacy as an overarching construct and distinguishes between General Teaching Efficacy (GTE) and Personal Teaching Efficacy (PTE). GTE concerns teachers’ general judgments about whether teaching can overcome external constraints and produce educational effects, whereas PTE concerns teachers’ judgments about their own ability to organize and implement teaching effectively. PTE is more closely aligned with personal efficacy beliefs in social cognitive theory, whereas GTE also encompasses broader outcome expectancies regarding the effectiveness of teaching. Although the two constructs are not psychologically identical, both involve judgments about the effectiveness and controllability of teaching (Bandura, 1997; Tschannen-Moran and Hoy, 2001; Jerrim et al., 2025). Higher teacher self-efficacy has generally been associated with more positive instructional practices, stronger work engagement and job satisfaction, and lower emotional exhaustion (Chen, 2025; Jerrim et al., 2025; Xu and Jia, 2022; Zee and Koomen, 2016).

Teaching efficacy does not develop independently of context. Teachers evaluate their personal capabilities and the effectiveness of teaching activities on the basis of prior teaching experiences, feedback from others, emotional states, and the perceived controllability of instructional tasks (Bandura, 1997; Morris et al., 2017). Job Demands-Resources theory defines job resources as working conditions that facilitate the attainment of work goals, reduce the costs associated with job demands, or promote individual growth (Demerouti et al., 2001; Bakker and Demerouti, 2017). In higher education, institutional safeguards, funding and equipment, task coordination, professional feedback, organizational recognition, and collegial collaboration may all function as job resources available to teachers (Bakker and Demerouti, 2017; Li et al., 2025a). These conditions can reduce practical barriers to teaching and provide a contextual basis for developing and maintaining positive efficacy judgments. They may increase opportunities for successful teaching, provide social feedback, and strengthen perceived task controllability.

As an important job resource closely related to teaching efficacy, teacher support is characterized by multiple sources and differentiated functions. In terms of source, universities provide organizational support through institutional arrangements, resource allocation, and professional development opportunities; department leaders provide proximal managerial support through task assignment, feedback, recognition, and problem coordination; and colleagues provide horizontal support through information sharing, emotional exchange, and instructional collaboration (Eisenberger et al., 1986; Kottke and Sharafinski, 1988; Ladd and Henry, 2000; Rhoades and Eisenberger, 2002). In terms of function, support can be divided into emotional support, which communicates care, respect, and recognition, and material support, which provides equipment, information, resources, and practical assistance (Cohen and Wills, 1985). Later research has proposed more differentiated functional taxonomies of social support. For example, Thomas and Hodges (2026) distinguish emotional support, social contact and companionship, belonging support, information and guidance support, and tangible support. The present study adopted the broader emotional–material distinction because it provides a parsimonious functional framework that can be applied consistently across organizational, leadership, and colleague sources. This operationalization is also consistent with the Chinese-context questionnaire framework used by Xiong (2023), thereby supporting the planned source × function structure. By crossing source and function, this study distinguishes six dimensions: Organizational Emotional Support (OES), Organizational Material Support (OMS), Leadership Emotional Support (LES), Leadership Material Support (LMS), Colleague Emotional Support (CES), and Colleague Material Support (CMS).

A review of the empirical literature shows that most previous studies have used variable-centered approaches to examine average associations between organizational, leadership, or colleague support and outcomes such as teaching efficacy, work engagement, and job satisfaction (Feng et al., 2026; Morris et al., 2017). Such research can determine whether a particular form of support is generally associated with teacher outcomes, but it is less able to reveal how multiple resources are jointly configured within individuals (Spurk et al., 2020). In practice, some teachers may receive high support from all sources, some may experience generally low support, and others may have adequate formal organizational resources but limited colleague collaboration, or limited formal organizational support but strong support from leaders and colleagues. Examining each source separately or calculating an overall support score may obscure groups of teachers with different source configurations.

Latent Profile Analysis is a typical person-centered approach that identifies subgroups characterized by within-group similarity and between-group differences on the basis of the joint distribution of multiple continuous indicators (Collins and Lanza, 2010; Masyn, 2013). Recent studies of teachers have identified distinct profiles of self-efficacy, psychological functioning, and job resources, and have found that these profiles differ in teacher well-being, instructional innovation, and work-related outcomes (Huang et al., 2026; Sun and Yin, 2025; Sun and Yuan, 2025). However, existing profile studies of teacher resources have generally focused on a limited set of resource levels or on overall job resources, and have rarely distinguished simultaneously among three sources—organization, leadership, and colleagues—and two functions—emotional and material support. Consequently, direct evidence remains limited regarding whether teacher support profiles are organized primarily by overall resource level, support source, or support function. Without such person-centered evidence, average associations may conceal configurations in which adequate support from one source coexists with substantial deficits in another. A profile-based approach can therefore clarify whether support heterogeneity primarily reflects overall resource adequacy or asymmetric source configurations, providing a more differentiated basis for understanding teachers’ support environments.

This issue is particularly relevant in the context of Chinese private universities. Compared with traditional public universities, private universities differ in governance structures, employment systems, resource provision, and faculty development mechanisms; accordingly, teachers may obtain formal institutional resources and informal relational support through different pathways (Qi, 2022; Zhang et al., 2021). Recent research in Chinese private higher education similarly indicates that quality of work life and perceived organizational support are important organizational resources associated with faculty retention-related outcomes (Zeng et al., 2025). Previous variable-centered research has shown that organizational, leadership, and colleague support are all positively associated with teaching efficacy among teachers in Chinese private universities, although the average strength of these associations differs across support sources (Feng et al., 2026). It remains unclear whether these differences merely reflect how much support teachers receive overall or whether they also reflect asymmetric combinations of formal organizational resources and proximal relational resources.

To address these gaps, the present study extended the five-university dataset used in Feng et al. (2026) by including one additional private university, resulting in a sample of 400 teachers from six private universities in Qingdao, China. Emotional and material support from the organization, leaders, and colleagues were used as latent profile indicators, and the BCH method was used to compare GTE and PTE across the resulting profiles. The study addressed three research questions. First, what statistically and substantively meaningful profiles of multisource support resources can be identified among teachers in Chinese private universities? Second, to what extent do the identified profiles reflect differences in overall resource level, support source, and support function? Third, do teachers in different support profiles differ in GTE and PTE? The study extends teacher support research from average associations involving individual resources to person-level configurations of multisource resources. It examines how support environments are associated with two forms of teaching efficacy and provides a basis for identifying teachers with different support needs.

## 2 Materials and methods

### 2.1 Participants and procedure

The study protocol was reviewed and approved by the Research Ethics Committee of the International Education Department, Qingdao Hengxing University of Science and Technology (Approval No. QHX/0409102025). The study used an anonymous online questionnaire and collected no directly identifiable personal information.

The research team first obtained institution-level permission through contacts in the academic affairs offices of the participating universities. Each university provided an official stamped letter confirming permission for its teachers to participate. Eligible participants were full-time teachers employed at one of the six participating private universities during the data-collection period. A proportionate stratified recruitment strategy was used, with each university constituting a stratum; sample allocations were determined approximately in proportion to each institution’s share of the combined faculty population across the six universities, with prespecified sex quotas to maintain approximately balanced representation of male and female teachers. Within universities, recruitment was conducted either with the assistance of authorized academic-affairs personnel or through on-site administration by the research team. Detailed institutional sample distributions are provided in Supplementary Table S21. The questionnaire was administered through Wenjuanxing, an online survey platform.

The first page of the questionnaire presented an informed consent statement explaining that the survey was for academic research only, participation was entirely voluntary, personal information would remain confidential, and participants could decline to respond or withdraw at any time. Participants could access the formal questionnaire only after providing informed consent. A total of 418 questionnaires were returned. Data-quality screening excluded responses if the 52-item questionnaire was completed in less than 2 minutes or if the same response option was selected across all Likert-type items, indicating complete straight-lining. Thirteen responses met the rapid-completion criterion and 16 met the complete straight-lining criterion, with 11 responses meeting both criteria. After accounting for this overlap, 18 responses were excluded, leaving 400 cases for analysis. There were no missing data on the core variables, all item responses fell within the valid range of 1–5, and no completely duplicate records were identified. To protect institutional confidentiality, the names of the six participating universities are not disclosed.

Of the 400 participants included in the present analysis, 334 (83.5%) were included in the five-university sample reported in Feng et al. (2026), whereas 66 participants (16.5%) from an additional sixth university were newly included. Accordingly, the present study constitutes an expanded secondary analysis of a partially overlapping dataset.

The final sample was evenly divided by sex, with men and women each accounting for 50.0%. In addition, 74.3% of participants were 40 years of age or younger, 67.2% held a master’s degree, 48.0% held the rank of lecturer or assistant research fellow, and 45.0% had 5 years or less of teaching experience. The number of valid respondents per university ranged from 12 to 112. Detailed demographic characteristics are presented in Table 1.

**Table 1:** Demographic characteristics of the sample (*N* = 400).

Variable	Category	Frequency	Percentage (%)
Sex	Male	200	50.0
	Female	200	50.0
Age	≤30 years	131	32.8
	31–40 years	166	41.5
	41–50 years	82	20.5
	≥51 years	21	5.2
Education	Doctorate	57	14.2
	Master’s	269	67.2
	Bachelor’s/other	74	18.5
Academic rank	Professor/Research Fellow	20	5.0
	Associate Professor/Associate Research Fellow	101	25.2
	Lecturer/Assistant Research Fellow	192	48.0
	Teaching Assistant/Research Intern	87	21.8
Teaching experience	0–5 years	180	45.0
	6–10 years	104	26.0
	11–15 years	50	12.5
	16–20 years	40	10.0
	≥21 years	26	6.5

### 2.2 Measures

Except for demographic information, all items were rated on 5-point Likert scales ranging from 1 (strongly disagree) to 5 (strongly agree), and each dimension was scored as the mean of its constituent items. The Chinese questionnaire battery was developed with reference to the original measurement instruments, subsequent Chinese adaptations, and the questionnaire used by Xiong (2023), and was further adapted for the context of teachers in Chinese private universities. Teaching efficacy was assessed using a measure based on the Teacher Efficacy Scale developed by Gibson and Dembo (1984), with reference to subsequent research on teaching efficacy in the Chinese context (Yu et al., 1995). The 14 retained items comprised General Teaching Efficacy (GTE; 6 items) and Personal Teaching Efficacy (PTE; 8 items). Organizational support was assessed using a measure adapted from the Survey of Perceived Organizational Support developed by Eisenberger et al. (1986), with reference to subsequent Chinese-context research on perceived organizational support (Chen and Liao, 2006). The 12 retained items comprised Organizational Emotional Support (OES; 6 items) and Organizational Material Support (OMS; 6 items). Leadership support was assessed using a measure based on the perceived supervisory support scale developed by Kottke and Sharafinski (1988). The 10 retained items comprised Leadership Emotional Support (LES; 5 items) and Leadership Material Support (LMS; 5 items). Colleague support was assessed using a measure based on the colleague support scale developed by Ladd and Henry (2000). The 10 retained items comprised Colleague Emotional Support (CES; 5 items) and Colleague Material Support (CMS; 5 items).

The questionnaire was further adapted for the teaching context of Chinese private universities through item selection and contextual wording adjustment. The item counts reported above refer to the final administered items. Complete final items, dimension assignments, and principal adaptation sources are provided in Supplementary Table S22. The adapted questionnaire underwent translation/back-translation, expert review, and pilot testing before formal administration. The LPA used full-sample z-standardized scores on the six support dimensions as profile indicators, with GTE and PTE treated as distal outcomes.

### 2.3 Data analysis

SPSS 26 was first used for data screening, descriptive statistics, Pearson correlations, and Cronbach’s α analyses. Mplus 9.1 was then used to estimate the confirmatory factor model, latent profile models, and BCH distal-outcome models. The eight-factor measurement model was estimated using WLSMV. Overall fit was evaluated using CFI, TLI, RMSEA, and SRMR, and standardized factor loadings, composite reliability, and average variance extracted were also reported. To examine discriminant validity among conceptually adjacent constructs, alternative models were estimated by merging GTE with PTE, OES with OMS, LES with LMS, and CES with CMS, respectively, and were compared using the WLSMV DIFFTEST procedure.

The LPA used the six z-standardized support dimensions as continuous indicators and sequentially estimated one- through seven-profile solutions. Although all six dimensions were scored on the same 1–5 metric, z-standardization was used to express deviations in common standard-deviation units and to facilitate comparison of profile shapes across indicators. In the baseline model, indicator means were allowed to vary across profiles, within-profile variances were constrained to equality, and within-profile covariances were fixed at 0. The latent profile models were estimated using robust maximum likelihood (MLR). To reduce the risk of convergence on local maxima, the random-start specification was set to STARTS = 16,000 3,200, meaning that 16,000 random start values were generated during the initial stage and the best 3,200 were retained for final-stage optimization. The maximum number of initial-stage iterations was set to STITERATIONS = 20. A solution was considered to have acceptable estimation stability when the best log-likelihood value was replicated across multiple random starts. Profile enumeration was based on AIC, BIC, aBIC, VLMR, Entropy, posterior classification probabilities, profile size, parameter identification, random-start stability, and substantive interpretability (Nylund et al., 2007).

After the profile solution had been selected, the BCH method was used to compare mean GTE and PTE across profiles while retaining classification uncertainty and reducing bias due to classification error (Asparouhov and Muthén, 2014; Vermunt, 2010). Pairwise *p* values were adjusted using the Holm procedure.

### 2.4 Robustness and sensitivity analyses

Several supplementary analyses were conducted to examine the robustness of the profile solution. First, the five-profile solution was retained as a more parsimonious competing solution. Second, the distribution of profiles across the six universities was examined. Third, within-profile residual correlations were allowed between the emotional and material dimensions of leadership support and colleague support. Fourth, LES and LMS were combined into an overall leadership support indicator, and the LPA was re-estimated using five indicators. Classification agreement between the primary and alternative models was evaluated using person-level agreement rates, Cohen’s κ, and the adjusted Rand index. Additional covariance-sensitivity models were estimated by freeing the within-profile covariance for each same-source emotional/material pair, including OES–OMS, and by considering a broader covariance specification. A further sensitivity analysis allowed indicator variances to differ across profiles while retaining zero within-profile covariances; five- and six-profile solutions were compared under this specification. Finally, the LPA was re-estimated using the original 1–5 dimension scores without standardization to evaluate sensitivity to indicator scaling.

## 3 Results

### 3.1 Preliminary analyses and measurement model

Preliminary data screening identified no severe univariate distributional departures. All six support dimensions were significantly and positively correlated with both GTE and PTE, with correlations ranging from 0.202 to 0.456 (all *p* < 0.001). The complete observed-score correlation matrix and skewness and kurtosis statistics for the six LPA indicators are reported in Supplementary Table S31.

Table 2 presents descriptive statistics, internal consistency, composite reliability, average variance extracted, and standardized factor loadings for the eight constructs. Cronbach’s α ranged from 0.690 to 0.920, and composite reliability ranged from 0.779 to 0.949, indicating generally acceptable internal consistency and construct reliability. Standardized factor loadings ranged from 0.472 to 0.903 and were all statistically significant. AVE exceeded 0.50 for OES, OMS, and CMS. The AVE values for GTE and CES were 0.492 and 0.476, respectively, which were close to 0.50; the corresponding values for LES and LMS were 0.452 and 0.415; and the AVE for PTE was 0.335. The latent correlation between LES and LMS was 0.886, and their HTMT value for LES and LMS was 0.872, further indicating substantial empirical overlap between the two leadership-support dimensions. Complete standardized item loadings are reported in Supplementary Table S3, and the latent-factor correlation and HTMT matrices are provided in Supplementary Tables S23, S24. Overall, the eight constructs demonstrated acceptable reliability, although item convergence was limited for some constructs, particularly PTE. Subsequent findings were therefore interpreted cautiously in conjunction with the overall model fit and evidence of discriminant validity.

**Table 2:** Descriptive statistics, reliability, and convergent validity of the study constructs (*N* = 400).

Construct	No. of items	M	SD	Cronbach’s α	CR	AVE	Standardized loading range
GTE	6	3.64	0.77	0.817	0.853	0.492	0.646–0.751
PTE	8	3.81	0.61	0.753	0.799	0.335	0.472–0.643
OES	6	3.77	0.65	0.898	0.932	0.696	0.773–0.874
OMS	6	3.91	0.67	0.920	0.949	0.755	0.847–0.903
LES	5	3.99	0.54	0.756	0.804	0.452	0.605–0.755
LMS	5	3.73	0.78	0.758	0.779	0.415	0.585–0.726
CES	5	4.16	0.38	0.690	0.819	0.476	0.620–0.731
CMS	5	3.87	0.65	0.810	0.854	0.545	0.514–0.815

M, mean; SD, standard deviation; CR, composite reliability; AVE, average variance extracted. CR, AVE, and standardized factor loadings were calculated from the STDYX-standardized estimates of the eight-factor confirmatory factor analysis.

The eight-factor measurement model showed good fit, χ^2^(961) = 1188.673, *p* < 0.001, CFI = 0.987, TLI = 0.986, RMSEA = 0.024, 90% CI [0.019, 0.029], SRMR = 0.043. These results indicated that the hypothesized eight-construct measurement structure fit the sample data well.

To further examine discriminant validity among conceptually adjacent constructs, four alternative seven-factor models were estimated by merging GTE with PTE, OES with OMS, LES with LMS, and CES with CMS, respectively. Merging GTE with PTE, OES with OMS, or CES with CMS significantly worsened model fit, supporting the discriminant validity of these constructs. By contrast, the difference between the seven-factor model that merged LES and LMS and the eight-factor model was not statistically significant, Δχ^2^(7) = 12.302, *p* = 0.091, indicating substantial empirical overlap between Leadership Emotional Support and Leadership Material Support. Despite this overlap, LES and LMS were retained as theoretically differentiated indicators in the primary LPA to preserve the planned source × function structure. Their separation was further evaluated in a sensitivity analysis using a combined leadership-support indicator.

### 3.2 Profile enumeration and model selection

Table 3 presents the fit indices for the one- through seven-profile solutions. AIC, BIC, and aBIC generally decreased as the number of profiles increased from one to seven, indicating improved relative model fit with additional profiles. Compared with the five-profile model, the six-profile model reduced AIC, BIC, and aBIC by 62.853, 34.912, and 57.124, respectively. However, Entropy declined slightly from 0.862 to 0.850, and the VLMR test for the six-profile solution was not statistically significant, *p* = 0.131. By contrast, the BLRT for the five- versus six-profile comparison favored the six-profile model, 2ΔLL = 76.852, *p* < 0.001. Thus, the likelihood-ratio tests provided mixed evidence regarding the additional sixth profile.

**Table 3:** Fit indices for the one- to seven-profile solutions.

No. of profiles	Free parameters	LL	AIC	BIC	aBIC	Entropy	VLMR *p*	BLRT p	Smallest profile
1	12	−3405.452	6834.905	6882.803	6844.726	–	–	–	400 (100.0%)
2	19	−3139.349	6316.698	6392.536	6332.248	0.826	<0.001	–	114 (28.5%)
3	26	−3027.979	6107.957	6211.735	6129.235	0.838	0.011	–	62 (15.5%)
4	33	−2973.296	6012.593	6144.311	6039.600	0.793	0.146	–	44 (11.0%)
5	40	−2928.410	5936.821	6096.479	5969.557	0.862	0.187	–	31 (7.75%)
**6**	**47**	**−2889.984**	**5873.968**	**6061.567**	**5912.433**	**0.850**	**0.131**	**< 0.001**	**21 (5.25%)**
7	54	−2867.571	5843.142	6058.681	5887.335	0.845	0.098	–	2 (0.50%)

The seven-profile model yielded a two-person profile and a nonidentification warning.

BLRT was additionally evaluated for the focal comparison between the five- and six-profile solutions; the six-profile model was favored over the five-profile model, 2ΔLL = 76.852, *p* < 0.001.

Bold values indicate the retained primary six-profile solution.

Model selection also considered profile size, solution stability, and substantive interpretability. Under a large random-start specification, the best log-likelihood value for the six-profile model was replicated and the model terminated normally, indicating acceptable estimation stability. Average posterior classification probabilities for the six profiles ranged from 0.865 to 0.931. The smallest profile contained 21 participants, representing 5.25% of the sample. Relative to the five-profile solution, the six-profile solution further differentiated the original low-support group into the Very Low Overall Support Profile and the Low Overall Support Profile. Both showed generally low support across the six indicators, but at different levels. GTE and PTE were not used in determining the number of profiles; BCH distal-outcome comparisons were conducted only after the profile solution had been selected. Although the seven-profile solution further reduced the information criteria, it produced a profile with only two participants and generated a nonidentification warning, suggesting possible overextraction. Considering the information criteria, BLRT, classification quality, profile size, estimation stability, and substantive interpretability together, the six-profile solution was retained as the primary representation of the present sample. However, given the nonsignificant VLMR and the slightly higher entropy of the five-profile model, the five-profile solution was retained as a plausible, more parsimonious competing representation.

### 3.3 The six support resource profiles

Profile labels were based on the z-standardized means of the six support indicators and their relative shapes. Profiles in which all indicators rose or fell together were labeled primarily in terms of overall support level, whereas profiles characterized by marked imbalances across support sources were labeled according to the dominant source. The labels describe relative patterns within the present sample and should not be interpreted as absolute diagnostic criteria or fixed teacher types. On this basis, the six profiles were labeled the Very Low Overall Support Profile, Low Overall Support Profile, Moderate Balanced Support Profile, Relational Support-Dominant Profile, Organizational Support-Dominant Profile, and High Balanced Support Profile.

The standardized means for the six profiles are shown in Table 4 and Figure 1. Four profiles primarily reflected differences in overall support level. The Very Low Overall Support Profile (5.25%) scored substantially below the sample mean on all six indicators. The Low Overall Support Profile (17.25%) also showed generally insufficient support, but to a lesser degree. The Moderate Balanced Support Profile (40.25%) scored close to the sample mean on all indicators. The High Balanced Support Profile (20.00%) scored above the sample mean on all six indicators.

**Table 4:** Profile sizes, classification quality, and standardized indicator means.

Profile	*n*	%	Post. prob.	OES	OMS	LES	LMS	CES	CMS
Very low overall support profile	21	5.25	0.919	−1.940	−1.766	−1.901	−1.815	−1.172	−1.146
Low overall support profile	69	17.25	0.865	−0.643	−0.720	−0.858	−1.149	−0.497	−0.792
Moderate balanced support profile	161	40.25	0.893	−0.066	−0.065	0.043	0.179	−0.270	−0.047
Relational support-dominant profile	32	8.00	0.892	−0.883	−0.900	0.423	0.286	1.143	1.099
Organizational support-dominant profile	37	9.25	0.931	1.518	1.499	0.643	0.581	−0.204	−0.899
High balanced support profile	80	20.00	0.895	0.861	0.895	0.688	0.728	0.902	1.039

All six indicators are full-sample z scores; Entropy = 0.850. Profile labels were based on relative profile shapes and do not represent absolute diagnostic cutoffs on the scales.

**Figure 1:**
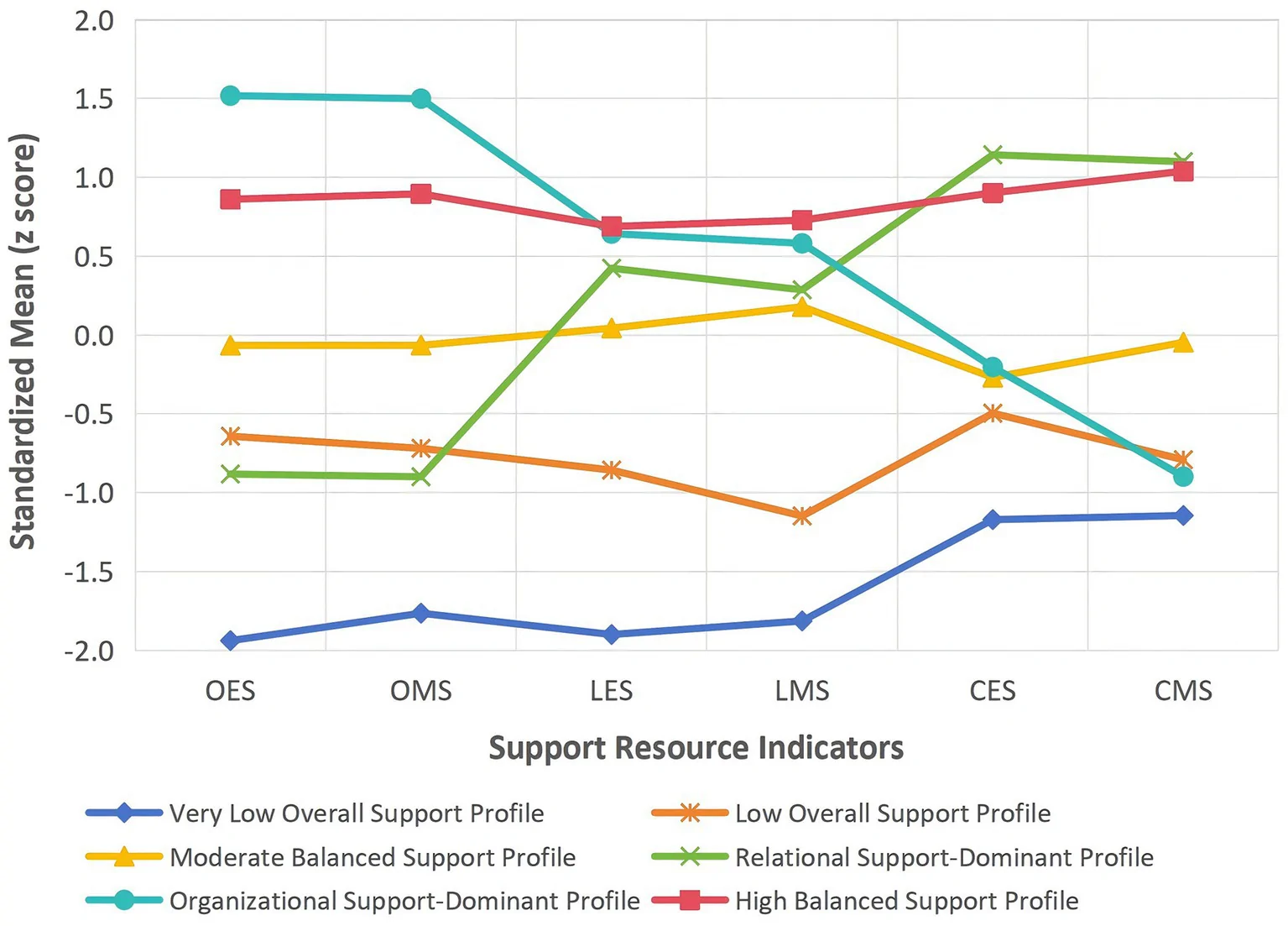
Six multisource support profiles. OES, Organizational Emotional Support; OMS, Organizational Material Support; LES, Leadership Emotional Support; LMS, Leadership Material Support; CES, Colleague Emotional Support; CMS, Colleague Material Support. All six indicators were z-standardized using the full sample. Profile names are descriptive, sample-relative labels and should not be interpreted as diagnostic classifications or fixed teacher types.

The remaining two profiles primarily reflected asymmetric configurations across support sources. In the Relational Support-Dominant Profile (8.00%), OES and OMS were both below the sample mean, whereas leadership and colleague support were relatively high. In the Organizational Support-Dominant Profile (9.25%), both organizational support indicators were substantially above the sample mean, whereas colleague support—particularly CMS—was relatively limited. At the same time, emotional and material support within the same source generally changed in similar directions across profiles, and no clear and stable emotional-support-dominant or material-support-dominant profile emerged. Thus, profile heterogeneity was organized mainly by overall support level and support-source structure, whereas independent differentiation by support function was relatively limited.

### 3.4 Differences in teaching efficacy across support profiles

As shown in Table 5, the overall BCH tests showed significant profile differences in GTE, χ^2^(5) = 131.378, *p* < 0.001, and in PTE, χ^2^(5) = 130.502, *p* < 0.001. The Very Low Overall Support Profile had the lowest mean on both dimensions of teaching efficacy, whereas the Organizational Support-Dominant Profile and the High Balanced Support Profile had relatively high means.

**Table 5:** BCH-adjusted means of teaching efficacy across the support profiles.

Profile	GTE M	SE	95% CI	PTE M	SE	95% CI
Very low overall support profile	2.663	0.164	[2.342, 2.984]	3.129	0.113	[2.908, 3.350]
Low overall support profile	3.245	0.099	[3.051, 3.440]	3.554	0.078	[3.401, 3.706]
Moderate balanced support profile	3.566	0.063	[3.442, 3.689]	3.703	0.048	[3.610, 3.797]
Relational support-dominant profile	3.717	0.147	[3.430, 4.005]	3.908	0.137	[3.639, 4.177]
Organizational support-dominant profile	4.193	0.096	[4.005, 4.381]	4.276	0.074	[4.130, 4.422]
High balanced support profile	4.105	0.078	[3.952, 4.257]	4.185	0.071	[4.046, 4.324]

Profile labels were based on the relative shapes of the six support indicators and do not represent absolute diagnostic classifications on the scales.

Overall test for GTE: χ^2^(5) = 131.378, *p* < 0.001; overall test for PTE: χ^2^(5) = 130.502, *p* < 0.001.

After Holm correction, the Very Low Overall Support Profile had significantly lower GTE and PTE than all other profiles. The Low Overall Support Profile and Moderate Balanced Support Profile both had lower scores than the Organizational Support-Dominant Profile and High Balanced Support Profile. The difference between the Very Low Overall Support Profile and the Low Overall Support Profile was also statistically significant after Holm correction. The Organizational Support-Dominant Profile and High Balanced Support Profile did not differ significantly in either GTE or PTE. The Relational Support-Dominant Profile had means between those of the Moderate Balanced Support Profile and the two high-efficacy profiles, but most pairwise comparisons were not statistically significant after correction for multiple comparisons. Complete BCH pairwise comparisons, including mean differences, standard errors, 95% confidence intervals, and Holm-adjusted *p* values, are reported in Supplementary Tables S8, S9.

### 3.5 Robustness and sensitivity analyses

The five-profile competing solution reproduced the Moderate Balanced Support Profile, Relational Support-Dominant Profile, Organizational Support-Dominant Profile, and High Balanced Support Profile. Its main difference was that it combined the Very Low Overall Support Profile and Low Overall Support Profile from the six-profile solution into a single overall low-support profile. The overall ordering of teaching efficacy in the five-profile model was consistent with that in the six-profile model, indicating that the principal conclusions did not depend entirely on further subdividing the low-support group. Thus, the broader support-level gradient and the two source-dominant configurations were common to both solutions, whereas the finer separation of the low-support end was specific to the six-profile representation.

When the OES–OMS within-profile covariance was freed separately, the model produced a two-person profile and a non-positive definite information matrix and was therefore treated as diagnostic only. A broader model freeing all three same-source emotional/material covariances also showed estimation instability. When within-profile residual correlations were allowed between the emotional and material dimensions of leadership support and colleague support, the model still identified a six-profile structure similar to the baseline solution. The person-level classification agreement between the local-dependence model and the baseline model was 89.0%, Cohen’s κ = 0.851, and the adjusted Rand index was 0.706, indicating high but not perfect agreement between the two solutions. When LES and LMS were combined into an overall leadership support indicator, the five-indicator model again identified six similar profiles. Agreement with the primary model was 94.25%, Cohen’s κ = 0.923, and the adjusted Rand index was 0.852. This high level of agreement indicates that the main profile structure was not primarily driven by modeling LES and LMS as separate indicators.

A further sensitivity analysis allowed indicator variances to differ across profiles while retaining zero within-profile covariances. The six-profile model was normally estimated and remained substantively interpretable (AIC = 5673.882, BIC = 5981.225, aBIC = 5736.899, Entropy = 0.854). Under the same variance specification, information criteria favored six profiles over five profiles. However, person-level classification agreement with the baseline equal-variance solution was 63.25% (Cohen’s κ = 0.526; adjusted Rand index = 0.373), indicating that exact profile boundaries and prevalence estimates were sensitive to the variance specification. The broader support-level gradient and the two source-dominant configurations remained recognizable across the alternative specification.

Descriptive inspection did not indicate that the smallest profile or either source-configuration profile was concentrated in a single university. Overall, the broader support-level gradient and source-dominant configurations were reproduced across alternative specifications, whereas exact profile boundaries, membership, and prevalence showed greater sensitivity when profile-specific variances were allowed. The selected six-profile solution should therefore be interpreted as a substantively informative representation of the present sample rather than as a fixed or uniquely determined classification.

## 4 Discussion

### 4.1 Main findings

The selected six-profile solution differentiated six patterns of multisource support in the present sample. Four profiles primarily reflected overall-level differences in which organizational, leadership, and colleague support rose or fell together, whereas two profiles reflected asymmetric configurations between formal organizational resources and more proximal sources of support from leaders and colleagues. By contrast, emotional and material support within the same source generally moved in the same direction, indicating that support function was not the primary dimension differentiating profiles in the present sample.

After the six-profile solution had been selected independently of the distal outcomes, BCH analyses showed significant differences in GTE and PTE across the resulting profiles. The Very Low Overall Support Profile had the lowest levels on both efficacy outcomes and scored significantly below the Low Overall Support Profile. The Organizational Support-Dominant Profile and High Balanced Support Profile had the highest means, with no significant difference between them. The five-profile competing model, the local-dependence model, the alternative model with a combined leadership support indicator, and the inspection of profile distributions across universities all broadly reproduced the primary structure. The six-profile solution can therefore be regarded as a well-supported representation of heterogeneity in support configurations in the current data, but it should not be interpreted as the only true or immutable classification of teachers.

### 4.2 Overall resource level as a potential primary organizing dimension of support heterogeneity

Four of the six profiles showed broadly parallel increases or decreases across all six indicators, suggesting that teacher support environments may first differentiate along an overall continuum of resource abundance. This finding is consistent with recent person-centered research on teachers’ job resources. Using multilevel Latent Profile Analysis, Li et al. (2025b) found that job demands, job resources, and personal resources among early childhood educators formed distinct configurations at both the educator and institutional levels and were systematically associated with multidimensional well-being. Pöysä et al. (2026) similarly found that profiles of teacher resilience and burnout included both level-based profiles in which psychological states were broadly favorable or unfavorable and configurations in which dimensions were not fully aligned. Together, these studies suggest that teachers’ job resources and psychological states often vary as relatively integrated patterns rather than as isolated dimensions.

The present study extends this pattern to teachers’ perceptions of support, showing that organizational, leadership, and colleague support may also vary as relatively integrated configurations rather than as isolated dimensions. One possible explanation is that support resources at different levels are interconnected in actual organizational processes (Bakker and Demerouti, 2017; Eisenberger et al., 1986; Rhoades and Eisenberger, 2002). For example, universities with better-developed institutional arrangements and more adequate resource allocation may also be more likely to foster supportive leadership practices and collegial collaboration. Conversely, limited institutional resources may constrain both the resources leaders can mobilize and the conditions under which colleagues can collaborate. From this perspective, the level-based profiles may reflect the clustering of support resources within the organizational system.

However, parallel movement across the six indicators does not necessarily mean that all six resources objectively increase or decrease together. Because all six support indicators were self-reported by the same teachers, the level-based profiles may also have been partly influenced by generalized evaluations of support and common method factors (Podsakoff et al., 2003). Teachers may first form an overall judgment about whether the university supports them and then extend that general evaluation to the organization, leaders, and colleagues. Accordingly, the present findings may reflect both the actual co-occurrence of resources and generalized perceptions of support or common method variance. Future research should combine institutional records, leader ratings, colleague reports, and objective resource indicators to distinguish actual resource co-occurrence from the alignment of perceived support.

The distinction between the Very Low Overall Support Profile and the Low Overall Support Profile is particularly noteworthy. Teachers in both profiles experienced generally insufficient support, but those in the Very Low Overall Support Profile reported even lower organizational, leadership, and colleague resources and significantly lower GTE and PTE. Li et al. (2025b) similarly showed that specific configurations of job and personal resources can identify groups with clearly different levels of well-being and cannot be reduced to the independent effect of any single resource. The present findings therefore suggest that moderate support insufficiency and extremely low support across multiple sources and both functions may represent substantively different work contexts.

Nevertheless, LPA summarizes continuous distributions into a finite number of profiles. The findings therefore do not establish that the Very Low Overall Support Profile represents an objectively existing threshold, nor do they demonstrate that teaching efficacy drops abruptly once support falls below a particular level. A more cautious interpretation is that the six-profile solution identified a subgroup at the lower end of the support continuum with particularly unfavorable teaching efficacy. Future research could use nonlinear regression, segmented models, or longitudinal latent transition analysis to determine whether the lowest levels of support are associated with genuinely cumulative or accelerating risks.

### 4.3 Configurations of formal organizational and relational resources

The emergence of the Organizational Support-Dominant Profile and the Relational Support-Dominant Profile indicates that teacher support environments are not organized solely along a single continuum from low to high support; they may also involve asymmetric configurations across support sources. The Organizational Support-Dominant Profile showed substantially above-average OES and OMS and above-average leadership support, but comparatively limited colleague support. By contrast, the Relational Support-Dominant Profile showed below-average organizational support but relatively high leadership and colleague support. The term Relational Support-Dominant Profile refers to relatively strong proximal interpersonal support from leaders and colleagues compared with formal organizational support (Zhang et al., 2025); it does not imply that leadership support lacks a formal managerial component. Both nonparallel profiles were identified in the primary and alternative models, suggesting that formal organizational resources and everyday proximal support can form distinct combinations.

The mean differences in GTE and PTE between the Organizational Support-Dominant Profile and the High Balanced Support Profile were not statistically significant. This pattern is broadly consistent with recent higher education research emphasizing the importance of organizational support. In a study of university teachers in western China, Chen (2025) found that perceived organizational support and teacher self-efficacy were significantly associated with work engagement and job satisfaction. Li L. et al. (2025) likewise reported significant relationships among perceived organizational support, self-efficacy, job satisfaction, and job performance among teachers in Chinese private universities. These variable-centered findings suggest that formal organizational support may provide an important contextual foundation for teaching efficacy judgments and other work outcomes.

The present study further shows that high organizational support does not necessarily coincide with high colleague support. Although the Organizational Support-Dominant Profile lacked the broad and balanced resource base of the High Balanced Support Profile, its GTE and PTE means were similar, and the differences between the two profiles were not statistically significant. This finding suggests a degree of similarity between the two configurations on the two forms of teaching efficacy examined here. However, a non-significant difference does not establish statistical equivalence and cannot be used to conclude that high organizational support substitutes for leadership or colleague support.

One possible explanation is that institutional arrangements, teaching resources, professional development opportunities, and organizational recognition play a foundational role in teaching activities. According to Job Demands-Resources theory, job resources can reduce task barriers, facilitate goal attainment, and promote individual development (Bakker and Demerouti, 2017). From a social cognitive perspective, the institutional and material conditions provided by the organization may also be associated with more positive efficacy judgments through increased opportunities for successful teaching, positive social feedback, and strengthened perceived control over instructional tasks (Bandura, 1997; Tschannen-Moran and Hoy, 2001). Thus, the relatively high teaching efficacy observed in the Organizational Support-Dominant Profile may not reflect direct substitution for other support sources; instead, formal organizational resources may create favorable conditions for completing teaching tasks and accumulating mastery experiences.

Nevertheless, the current findings only indicate that the Organizational Support-Dominant Profile and High Balanced Support Profile did not differ significantly in GTE or PTE; they cannot be generalized to other teacher outcomes. Jensen et al. (2025) found that leadership support was associated with teacher self-efficacy and the emotional classroom climate, but the pattern was not identical across efficacy dimensions and student outcomes. This finding suggests that even when support from a particular source is strongly associated with a specific instructional judgment, it cannot be assumed to have equal value for all teacher outcomes. The Organizational Support-Dominant Profile and High Balanced Support Profile may still differ in knowledge sharing, instructional innovation, collaborative behavior, burnout, or turnover intentions, none of which were examined in the present study.

The Relational Support-Dominant Profile represents another resource configuration. Although teachers in this profile perceived relatively low formal organizational support, leadership and colleague support were comparatively strong, and their mean GTE and PTE remained at moderate-to-high levels. Malmström and Öqvist (2025) found that teachers’ perceived social support, self-efficacy, and motivation were jointly associated with teacher leadership behavior, suggesting that relational support is not merely a source of general emotional comfort but may also be embedded in teachers’ professional action and motivational processes. Chen and Rong (2023) similarly found that teacher collegiality among Chinese teachers may provide information exchange, professional collaboration, and support for action. Jensen et al. (2025) further indicated that feedback and support from leaders may be associated with teachers’ efficacy judgments in specific instructional tasks.

It is important to note that, after Holm correction, most pairwise differences between the Relational Support-Dominant Profile and the Organizational Support-Dominant and High Balanced Support Profiles were not statistically significant. The present findings therefore neither establish a stable ordering of efficacy across these three resource configurations nor demonstrate that leadership and colleague support fully compensate for limited formal organizational resources. A cautious interpretation is that, when formal organizational resources are relatively limited, proximal support from leaders and colleagues may be associated with maintaining relatively positive teaching efficacy; however, such support should not be regarded as a complete substitute for institutional safeguards, funding and equipment, or professional development opportunities. Relational support may also depend on particular leaders, colleague networks, and everyday interaction contexts. If leaders change or collaboration networks are disrupted, relational support may be less stable than institutionalized support. Because the present study did not directly test the temporal stability of support configurations, these interpretations require further examination using longitudinal and multisource data.

### 4.4 Potential bidirectional processes between support configurations and teaching efficacy

The highly similar ordering of the six profiles on GTE and PTE indicates that multisource support configurations are associated with both teachers’ judgments of their personal instructional capability and their general beliefs about whether teaching can produce the intended educational effects. Recent research provides additional evidence for links among environmental support, efficacy judgments, emotional experiences, and teacher engagement.

In the context of online teaching in China, Liu et al. (2025) found that institutional support was significantly associated with teacher engagement and that digital self-efficacy and negative emotions formed corresponding indirect pathways. Chen (2025) also reported that organizational support, self-efficacy, and work engagement were jointly associated with job satisfaction among university teachers. These studies suggest that support environments provide practical resources and also operate in conjunction with teachers’ capability judgments and emotional experiences.

However, like the present study, these studies relied primarily on cross-sectional questionnaires and theoretically specified path models. Their indirect or mediating effects therefore indicate that the data are compatible with a proposed mechanism but do not establish that changes in organizational support temporally precede changes in efficacy. Similarly, the profile differences observed in the present study cannot be interpreted as direct evidence that support resources increased or decreased teaching efficacy.

Conversely, the present findings cannot rule out reverse selection or reciprocal processes. Although Burić et al. (2020) did not directly examine support resources, their longitudinal findings indicate that teacher efficacy may contribute to shaping subsequent work experiences. More directly, Xanthopoulou et al. (2009) found possible reciprocal relationships between job resources and personal psychological resources. This evidence leaves open the possibility that teachers with higher efficacy are more proactive in seeking resources, participating in collaboration, and obtaining organizational recognition. Thus, the higher efficacy observed in the Organizational Support-Dominant and High Balanced Support Profiles may reflect both the influence of resource-rich environments on teachers’ judgments and the greater likelihood that highly efficacious teachers obtain or actively construct resource-rich work environments.

The same bidirectional interpretation applies to the Relational Support-Dominant Profile. Leadership and colleague support may help teachers maintain efficacy, but teachers with higher efficacy, stronger communication skills, or greater willingness to collaborate may also be more likely to establish stable relational support networks. Support configurations and teaching efficacy may therefore reinforce one another rather than operate through a simple unidirectional process. Future research should use multiwave cross-lagged models, latent transition analysis, or resource-based interventions (e.g., Täschner et al., 2025) to test whether changes in support profiles precede changes in efficacy and whether efficacy, in turn, predicts transitions into more favorable support profiles.

The similar ordering of GTE and PTE may also partly reflect measurement overlap. Although the measurement model supported the distinction between the two constructs, they were highly correlated, and PTE showed relatively limited convergent validity. The parallel gradients in the two efficacy measures may therefore have theoretical meaning but may also be influenced by their common reporting source and similarities in item content. Future studies should incorporate classroom observations, student evaluations, and actual instructional behavior to determine whether multisource support configurations are also associated with multisource or objective teaching outcomes.

### 4.5 Theoretical and practical implications

The study has three main theoretical implications.

First, the findings indicate that heterogeneity in multisource teacher support is organized primarily by overall resource level, whereas independent differentiation by support function is less pronounced. Four of the six profiles showed broadly parallel changes in the emotional and material dimensions of organizational, leadership, and colleague support, suggesting that teacher support environments first differentiate according to overall resource adequacy. This pattern is consistent with recent person-centered studies of job resources and teacher psychological characteristics, in which multiple resources or psychological dimensions often cluster into generally high, moderate, or low patterns (Li et al., 2025b; Pöysä et al., 2026). The present study further shows that, even after distinguishing three sources and two functions, overall resource level remains the primary structural feature of teacher support profiles, whereas emotional and material support within the same source do not form stable and independent function-dominant profiles.

Second, the findings suggest that different support sources may show limited functional similarity for specific outcomes. The Organizational Support-Dominant Profile and High Balanced Support Profile did not differ significantly in GTE or PTE, indicating similar patterns across the two outcomes examined here. However, nonsignificant differences do not establish statistical equivalence and therefore do not demonstrate that high organizational support can substitute for leadership or colleague support. Job Demands-Resources theory proposes that job resources reduce task barriers and facilitate goal attainment, whereas social cognitive theory suggests that environmental conditions may be associated with efficacy judgments through increased opportunities for success, positive feedback, and strengthened task controllability (Bakker and Demerouti, 2017; Bandura, 1997; Tschannen-Moran and Hoy, 2001). Nevertheless, similarity on the present outcomes cannot be generalized into a claim that organizational support broadly replaces leadership or colleague support. Different sources may vary in the resources they control, their temporal stability, and their effects on knowledge sharing, collaborative behavior, and emotional experience (Eisenberger et al., 1986; Jensen et al., 2025; Ladd and Henry, 2000).

Third, the study further differentiates heterogeneity within the overall low-support group. The five-profile solution combined the Very Low Overall Support Profile and Low Overall Support Profile, whereas the six-profile solution distinguished them and showed that the Very Low Overall Support Profile had significantly lower GTE and PTE. In the baseline six-profile solution, the lower end of the support continuum was further differentiated into Very Low Overall and Low Overall Support patterns, with the former showing lower GTE and PTE. However, this finer subdivision was less stable under alternative variance specifications and should not be interpreted as evidence of an objective threshold or qualitatively distinct low-support types. Traditional variable-centered analyses generally estimate average linear associations between support variables and teacher outcomes and are less able to identify person-level patterns characterized by simultaneous resource deficits. Person-centered analyses can instead reveal configurations that share the same general direction but differ in degree (Li et al., 2025b; Spurk et al., 2020). However, this finding does not establish an objective risk threshold. Longitudinal and nonlinear research is needed to determine whether extremely low support has cumulative or accelerating consequences (Masyn, 2013).

At the practical level, private universities could use multidimensional support-needs assessments rather than relying on a single total support score. The present profiles should not be used as diagnostic categories or classification cutoffs for individual teachers; instead, they highlight dimensions and combinations of support that may warrant attention. When assessment indicates broadly low support across multiple sources, attention may be directed toward simultaneous deficiencies in institutional resources, task coordination, leadership feedback, and colleague collaboration. When organizational resources are relatively strong but colleague support is comparatively limited, universities may consider strengthening knowledge sharing and instructional collaboration. Conversely, when proximal leadership and colleague support are relatively strong but formal organizational resources are limited, effective relational practices may inform improvements in more stable institutional arrangements and resource channels. These implications are intended to guide support planning rather than to prescribe profile-specific interventions, whose effectiveness was not tested in the present study.

These managerial implications should nevertheless be interpreted cautiously. Recent studies have primarily established statistical associations among support, self-efficacy, engagement, and teacher behavior (Chen, 2025; Jensen et al., 2025; Li L. et al., 2025; Malmström and Öqvist, 2025), but they do not demonstrate that profile-specific interventions will necessarily produce the intended effects. Latent profiles are therefore better suited to describing resource configurations and informing support planning than to serving as fixed classifications of individual teachers (Masyn, 2013; Spurk et al., 2020).

### 4.6 Limitations and future directions

This study has several limitations. First, the cross-sectional self-report design cannot establish the temporal ordering or causal direction between support configurations and teaching efficacy and may be affected by common method bias. Previous longitudinal research suggests that job resources and personal psychological resources may influence one another (Xanthopoulou et al., 2009). Future research should use multiwave longitudinal designs, latent transition analysis, or intervention studies to test whether changes in support profiles precede changes in teaching efficacy and whether teaching efficacy, in turn, predicts transitions into different support profiles.

Second, the sample was drawn from only six private universities in Qingdao, China. The profile structure and profile proportions therefore require replication in other regions, public universities, and different disciplinary groups. Some profiles were small, which may reduce the stability of their estimated means and distal-outcome comparisons. In addition, teachers were nested within six universities, but university-level clustering was not modeled. The small number of universities was also insufficient for stable estimation of a multilevel latent profile model. Although the observed ICCs were low (≤ 0.033) and the profile cross-tabulation showed no marked concentration within individual universities, these findings are descriptive given the small number of institutional clusters and do not rule out university-level contextual effects. Future studies with a larger number of institutions should examine how university-level institutional environments shape profile structures.

Third, the measurement quality of several constructs was limited. Although the overall eight-factor measurement model fit well, convergent validity was relatively limited for PTE, LES, and LMS, and LES and LMS showed substantial empirical overlap. Although the alternative model using a combined leadership support indicator broadly reproduced the primary profile structure, the constructs and the resulting profile interpretations require further validation in independent samples.

Fourth, the distal outcomes were limited to teachers’ self-reported teaching efficacy. Future research should incorporate classroom observations, student evaluations, instructional innovation, work engagement, burnout, and turnover intentions to determine whether the Organizational Support-Dominant Profile and High Balanced Support Profile remain similar across a broader range of outcomes.

Fifth, the study focused on the configuration of multisource support resources and their associations with teaching efficacy and did not further examine whether age, teaching experience, academic rank, or educational attainment predicted profile membership. It therefore remains possible that teachers at different career stages are more likely to enter particular support profiles. Future research could examine these demographic characteristics as profile predictors.

## 5 Conclusion

This study shows that understanding teacher support requires attention not only to the amount of support available, but also to how resources from different sources are combined within teachers’ work environments. A person-centered perspective revealed that multisource support cannot be fully represented by separate average associations or by a single overall support score. Instead, formal organizational resources and more proximal relational resources may coexist in different configurations, with these configurations corresponding to meaningful differences in teachers’ efficacy beliefs. The findings therefore extend existing variable-centered research by highlighting the configurational nature of support environments in Chinese private universities. From an applied perspective, support assessment may be more informative when it considers patterns of resource adequacy across organizational, leadership, and colleague sources rather than treating support as a uniform construct. At the same time, the identified profiles should be viewed as sample-specific representations of resource patterns rather than fixed categories of teachers. Future longitudinal and multisource research is needed to examine how these configurations develop, change over time, and relate to actual teaching processes and outcomes.

## Data Availability

The datasets presented in this article are not readily available because the data supporting the findings of this study are available from the author upon reasonable request. Requests to access the datasets should be directed to HF, fenghuimin@qdhxu.edu.cn.
