# Supplementary material for: Profiles of multisource support and teaching efficacy among teachers in Chinese private universities: a latent profile analysis
Source: Front Psychol. 2026 Sep 28;17:1961537. doi: 10.3389/fpsyg.2026.1961537 (PMC13655021; doi:10.3389/fpsyg.2026.1961537)
Supplement: Supplementary file 1 [file Data_Sheet_1.PDF]

Supplementary Material

This supplement documents the measurement-model comparisons, profile enumeration, BCH distal-outcome analyses, Holm-corrected pairwise comparisons, and robustness/sensitivity analyses reported in the manuscript. Statistics were extracted from the final SPSS and Mplus outputs. Holm-adjusted p values were computed separately for the 15 pairwise comparisons for each six-profile distal outcome.

1. Measurement-Model Verification

Supplementary Table S1. Fit comparison of the eight-factor measurement model and seven-factor alternatives

Model	χ^2	df	CFI	TLI	RMSEA	RMSEA 90% CI	SRMR	WLSMV DIFFTEST
Eight-factor baseline	1188.673	961	.987	.986	.024	[.019, .029]	.043	—
GTE and PTE merged	1220.122	968	.986	.985	.026	[.021, .030]	.045	$\Delta\chi^2(7) = 23.049$, $p = .0017$
OES and OMS merged	1355.355	968	.978	.976	.032	[.028, .036]	.046	$\Delta\chi^2(7) = 91.527$, $p < .001$
LES and LMS merged	1199.642	968	.987	.986	.024	[.020, .029]	.044	$\Delta\chi^2(7) = 12.302$, $p = .0911$
CES and CMS merged	1280.931	968	.982	.981	.028	[.024, .033]	.046	$\Delta\chi^2(7) = 46.681$, $p < .001$

Note. DIFFTEST values are WLSMV-adjusted chi-square difference tests against the eight-factor baseline model. The LES/LMS merged model did not fit significantly worse than the baseline model, indicating substantial empirical overlap between LES and LMS.

Supplementary Table S2. Construct-level descriptive statistics, reliability, and convergent-validity indices

Construct	Items	M	SD	Cronbach's α	CR	AVE	STDYX loading range
GTE	6	3.64	.77	.817	.853	.492	.646-.751
PTE	8	3.81	.61	.753	.799	.335	.472-.643
OES	6	3.77	.65	.898	.932	.696	.773-.874
OMS	6	3.91	.67	.920	.949	.755	.847-.903
LES	5	3.99	.54	.756	.804	.452	.605-.755
LMS	5	3.73	.78	.758	.779	.415	.585-.726
CES	5	4.16	.38	.690	.819	.476	.620-.731
CMS	5	3.87	.65	.810	.854	.545	.514-.815

Note. CR, AVE, and standardized loading ranges were calculated from the STDYX solution of the eight-factor WLSMV model. All standardized loadings were statistically significant.

Supplementary Table S3. Item-level standardized factor loadings for the eight-factor model

Construct	Item	STDYX loading	Construct	Item	STDYX loading
GTE	B1	.671	OMS	C10	.847
GTE	B2	.662	OMS	C11	.864
GTE	B3	.751	OMS	C12	.884
GTE	B4	.646	LES	D1	.626
GTE	B5	.722	LES	D2	.696
GTE	B6	.748	LES	D3	.669
PTE	B7	.643	LES	D4	.605
PTE	B8	.523	LES	D5	.755
PTE	B9	.636	LMS	D6	.585
PTE	B10	.472	LMS	D7	.600
PTE	B11	.633	LMS	D8	.657
PTE	B12	.572	LMS	D9	.726
PTE	B13	.595	LMS	D10	.642
PTE	B14	.532	CES	E1	.731
OES	C1	.773	CES	E2	.639
OES	C2	.811	CES	E3	.720
OES	C3	.835	CES	E4	.731
OES	C4	.859	CES	E5	.620
OES	C5	.851	CMS	E6	.758
OES	C6	.874	CMS	E7	.800
OMS	C7	.867	CMS	E8	.815
OMS	C8	.847	CMS	E9	.514
OMS	C9	.903	CMS	E10	.764

Note. All loadings were significant at $p < .001$. Item labels correspond to the anonymized item codes used in the analysis files.

2. Baseline Profile Enumeration and Six-Profile Solution

Estimation settings. The baseline LPA used six z-standardized continuous indicators and MLR estimation. For the final six-profile check, STARTS = 16000 3200 and STITERATIONS = 20. The best loglikelihood value was replicated and the model terminated normally.

Supplementary Table S4. Enumeration results for one- through seven-profile solutions

Profiles	LL	AIC	BIC	aBIC	Entropy	VLMR p	Smallest n	Smallest %
1	-3405.452	6834.905	6882.803	6844.726	—	—	400	100.00
2	-3139.349	6316.698	6392.536	6332.248	.826	< .001	114	28.50
3	-3027.979	6107.957	6211.735	6129.235	.838	.011	62	15.50
4	-2973.296	6012.593	6144.311	6039.600	.793	.146	44	11.00
5	-2928.410	5936.821	6096.479	5969.557	.862	.187	31	7.75
6	-2889.984	5873.968	6061.567	5912.433	.850	.131	21	5.25
7	-2867.571	5843.142	6058.681	5887.335	.845	.098	2	0.50

Note. The seven-profile solution produced a two-person profile and a non-positive definite information-matrix warning. The six-profile solution was retained on the basis of information criteria, classification quality, profile size, estimation stability, and substantive interpretability.

Supplementary Table S5. Baseline six-profile solution: size, classification quality, and standardized indicator means

Profile	Mplus class	n	%	Posterior probability	OES	OMS	LES	LMS	CES	CMS
Very Low Overall Support Profile	1	21	5.25	.919	-1.940	-1.766	-1.901	-1.815	-1.172	-1.146
Low Overall Support Profile	3	69	17.25	.865	-.643	-.720	-.858	-1.149	-.497	-.792
Moderate Balanced Support Profile	2	161	40.25	.893	-.066	-.065	.043	.179	-.270	-.047
Relational Support-Dominant Profile	5	32	8.00	.892	-.883	-.900	.423	.286	1.143	1.099
Organizational Support-Dominant Profile	4	37	9.25	.931	1.518	1.499	.643	.581	-.204	-.899
High Balanced Support Profile	6	80	20.00	.895	.861	.895	.688	.728	.902	1.039

Note. Indicators are full-sample z scores. Profile labels are descriptive, sample-relative labels rather than absolute diagnostic categories.

Supplementary Table S6. Average posterior probabilities for most-likely profile membership in the baseline six-profile solution

Most-likely class	Class 1	Class 2	Class 3	Class 4	Class 5	Class 6
Mplus class 1	.919	.000	.081	.000	.000	.000
Mplus class 2	.000	.893	.046	.004	.019	.038
Mplus class 3	.025	.109	.865	.000	.002	.000
Mplus class 4	.000	.011	.000	.931	.000	.058
Mplus class 5	.000	.075	.018	.000	.892	.015
Mplus class 6	.000	.072	.000	.020	.013	.895

Note. Rows represent most-likely class membership and columns represent estimated latent classes. Diagonal values range from .865 to .931.

3. Distal-Outcome Verification for the Baseline Six-Profile Solution

Supplementary Table S7. BCH-adjusted means of teaching efficacy outcomes

Profile	Mplus class	GTE M	GTE SE	GTE 95% CI	PTE M	PTE SE	PTE 95% CI
Very Low Overall Support Profile	1	2.663	.164	[2.342, 2.984]	3.129	.113	[2.908, 3.350]
Low Overall Support Profile	3	3.245	.099	[3.051, 3.440]	3.554	.078	[3.401, 3.706]
Moderate Balanced Support Profile	2	3.566	.063	[3.442, 3.689]	3.703	.048	[3.610, 3.797]
Relational Support-Dominant Profile	5	3.717	.147	[3.430, 4.005]	3.908	.137	[3.639, 4.177]
Organizational Support-Dominant Profile	4	4.193	.096	[4.005, 4.381]	4.276	.074	[4.130, 4.422]
High Balanced Support Profile	6	4.105	.078	[3.952, 4.257]	4.185	.071	[4.046, 4.324]

Note. GTE overall Wald test: $\chi^2(5) = 131.378$, $p < .001$. PTE overall Wald test: $\chi^2(5) = 130.502$, $p < .001$.

Supplementary Table S8. Full BCH pairwise comparisons for GTE with Holm correction

Profile A	Profile B	Mean difference (A−B)	SE	95% CI lower	95% CI upper	95% CI	Holm-adjusted p	Significant after Holm
Very Low Overall Support Profile	Moderate Balanced Support Profile	-0.903	0.175	-1.245	-0.560	[-1.245, -0.560]	< .001	Yes
Very Low Overall Support Profile	Low Overall Support Profile	-0.582	0.200	-0.973	-0.190	[-0.973, -0.190]	.025	Yes
Very Low Overall Support Profile	Organizational Support-Dominant Profile	-1.530	0.190	-1.902	-1.158	[-1.902, -1.158]	< .001	Yes
Very Low Overall Support Profile	Relational Support-Dominant Profile	-1.054	0.220	-1.485	-0.623	[-1.485, -0.623]	< .001	Yes
Very Low Overall Support Profile	High Balanced Support Profile	-1.442	0.181	-1.797	-1.086	[-1.797, -1.086]	< .001	Yes
Moderate Balanced Support Profile	Low Overall Support Profile	0.321	0.126	0.074	0.567	[0.074, 0.567]	.043	Yes
Moderate Balanced Support Profile	Organizational Support-Dominant Profile	-0.627	0.115	-0.853	-0.402	[-0.853, -0.402]	< .001	Yes
Moderate Balanced Support Profile	Relational Support-Dominant Profile	-0.151	0.164	-0.473	0.171	[-0.473, 0.171]	.714	No
Moderate Balanced Support Profile	High Balanced Support Profile	-0.539	0.105	-0.746	-0.332	[-0.746, -0.332]	< .001	Yes
Low Overall Support Profile	Organizational Support-Dominant Profile	-0.948	0.138	-1.219	-0.678	[-1.219, -0.678]	< .001	Yes
Low Overall Support Profile	Relational Support-Dominant Profile	-0.472	0.178	-0.820	-0.124	[-0.820, -0.124]	.039	Yes
Low Overall Support Profile	High Balanced Support Profile	-0.860	0.125	-1.106	-0.614	[-1.106, -0.614]	< .001	Yes
Organizational Support-Dominant Profile	Relational Support-Dominant Profile	0.476	0.175	0.133	0.819	[0.133, 0.819]	.039	Yes
Organizational Support-Dominant Profile	High Balanced Support Profile	0.088	0.128	-0.163	0.339	[-0.163, 0.339]	.714	No
Relational Support-Dominant Profile	High Balanced Support Profile	-0.388	0.168	-0.717	-0.058	[-0.717, -0.058]	.063	No

Note. Mean differences, standard errors, and 95% confidence intervals are taken directly from the BCH Mplus model for GTE. The 95% CI corresponds to the Mplus Lower 2.5% and Upper 2.5% limits. Holm adjustment was applied across the 15 pairwise tests for GTE. A nonsignificant comparison does not establish statistical equivalence.

Supplementary Table S9. Full BCH pairwise comparisons for PTE with Holm correction

Profile A	Profile B	Mean difference (A−B)	SE	95% CI lower	95% CI upper	95% CI	Holm-adjusted p	Significant after Holm
Very Low Overall Support Profile	Moderate Balanced Support Profile	-0.574	0.122	-0.813	-0.336	[-0.813, -0.336]	< .001	Yes
Very Low Overall Support Profile	Low Overall Support Profile	-0.425	0.143	-0.705	-0.145	[-0.705, -0.145]	.021	Yes
Very Low Overall Support Profile	Organizational Support-Dominant Profile	-1.147	0.135	-1.412	-0.883	[-1.412, -0.883]	< .001	Yes
Very Low Overall Support Profile	Relational Support-Dominant Profile	-0.779	0.177	-1.127	-0.432	[-1.127, -0.432]	< .001	Yes
Very Low Overall Support Profile	High Balanced Support Profile	-1.056	0.133	-1.317	-0.795	[-1.317, -0.795]	< .001	Yes
Moderate Balanced Support Profile	Low Overall Support Profile	0.149	0.097	-0.042	0.340	[-0.042, 0.340]	.377	No
Moderate Balanced Support Profile	Organizational Support-Dominant Profile	-0.573	0.089	-0.747	-0.399	[-0.747, -0.399]	< .001	Yes
Moderate Balanced Support Profile	Relational Support-Dominant Profile	-0.205	0.149	-0.497	0.087	[-0.497, 0.087]	.377	No
Moderate Balanced Support Profile	High Balanced Support Profile	-0.482	0.090	-0.658	-0.306	[-0.658, -0.306]	< .001	Yes
Low Overall Support Profile	Organizational Support-Dominant Profile	-0.722	0.108	-0.933	-0.511	[-0.933, -0.511]	< .001	Yes
Low Overall Support Profile	Relational Support-Dominant Profile	-0.354	0.158	-0.664	-0.045	[-0.664, -0.045]	.124	No
Low Overall Support Profile	High Balanced Support Profile	-0.631	0.105	-0.836	-0.426	[-0.836, -0.426]	< .001	Yes
Organizational Support-Dominant Profile	Relational Support-Dominant Profile	0.368	0.156	0.063	0.673	[0.063, 0.673]	.109	No
Organizational Support-Dominant Profile	High Balanced Support Profile	0.091	0.107	-0.119	0.301	[-0.119, 0.301]	.396	No
Relational Support-Dominant Profile	High Balanced Support Profile	-0.277	0.156	-0.583	0.029	[-0.583, 0.029]	.304	No

Note. Mean differences, standard errors, and 95% confidence intervals are taken directly from the BCH Mplus model for PTE. The 95% CI corresponds to the Mplus Lower 2.5% and Upper 2.5% limits. Holm adjustment was applied across the 15 pairwise tests for PTE. A nonsignificant comparison does not establish statistical equivalence.

4. Five-Profile Competing Solution

Estimation settings. The original six indicators were retained, but the number of profiles was fixed at five. MLR estimation used STARTS = 10000 2000 and STITERATIONS = 20. The best loglikelihood value was replicated and the model terminated normally. LL = -2928.410, AIC = 5936.821, BIC = 6096.479, aBIC = 5969.557, and Entropy = .862.

Supplementary Table S10. Original six-indicator five-profile competing solution

Profile	Mplus class	n	%	Posterior probability	OES	OMS	LES	LMS	CES	CMS
Low Overall Support Profile	1	56	14.00	.914	-1.234	-1.256	-1.391	-1.625	-.857	-1.014
Moderate Balanced Support Profile	2	182	45.50	.932	-.172	-.177	-.128	-.034	-.302	-.206
Organizational Support-Dominant Profile	3	35	8.75	.934	1.508	1.491	.656	.600	-.213	-.902
High Balanced Support Profile	4	96	24.00	.877	.795	.833	.694	.719	.799	.988
Relational Support-Dominant Profile	5	31	7.75	.882	-.830	-.855	.427	.286	1.194	1.147

Note. The five-profile solution reproduced the Moderate Balanced, Relational Support-Dominant, Organizational Support-Dominant, and High Balanced profiles, while representing the lower end of the support continuum as one broad Low Overall Support Profile.

Supplementary Table S11. BCH-adjusted teaching-efficacy means for the five-profile competing solution

Profile	Mplus class	GTE M	GTE SE	GTE 95% CI	PTE M	PTE SE	PTE 95% CI
Low Overall Support Profile	1	2.852	.099	[2.658, 3.046]	3.302	.076	[3.153, 3.451]
Moderate Balanced Support Profile	2	3.533	.056	[3.423, 3.643]	3.688	.043	[3.604, 3.771]
Organizational Support-Dominant Profile	3	4.212	.100	[4.015, 4.408]	4.244	.076	[4.096, 4.393]
High Balanced Support Profile	4	4.066	.074	[3.922, 4.211]	4.172	.066	[4.043, 4.301]
Relational Support-Dominant Profile	5	3.770	.149	[3.477, 4.062]	3.911	.145	[3.626, 4.196]

Note. GTE overall Wald test: $\chi^2(4) = 139.064$, $p < .001$. PTE overall Wald test: $\chi^2(4) = 122.185$, $p < .001$. The ordering of the profile means was consistent with the primary six-profile interpretation.

Supplementary Table S12. Profile-shape correspondence between the five- and six-profile solutions

Five-profile solution	Defining pattern	Closest six-profile counterpart	Interpretation
Five-profile Low Overall Support	Broad low-support pattern	Six-profile Very Low Overall + Low Overall	The six-profile solution separated the lower end into two severity levels.
Five-profile Moderate Balanced	Near-zero balanced pattern	Six-profile Moderate Balanced	Profile shape retained.
Five-profile Organizational Support-Dominant	High OES/OMS; lower colleague support	Six-profile Organizational Support-Dominant	Profile shape retained.
Five-profile High Balanced	High across all six indicators	Six-profile High Balanced	Profile shape retained.
Five-profile Relational Support-Dominant	Low OES/OMS; high leadership/colleague support	Six-profile Relational Support-Dominant	Profile shape retained.

Note. This table summarizes correspondence by estimated profile shape; it is not a case-level cross-classification table.

5. Local-Dependence Sensitivity Analyses

Supplementary Table S13. Comparison of local-dependence sensitivity models

Model	Specification	LL	AIC	BIC	aBIC	Entropy	Smallest n	Assessment
Baseline six-profile	Within-profile covariances fixed at 0	-2889.984	5873.968	6061.567	5912.433	.850	21	Acceptable baseline
OES WITH OMS	Organizational indicator residual correlation freed	-2866.520	5829.039	6020.629	5868.322	.803	2	Diagnostic only: two-person profile; non-positive definite information matrix
LES WITH LMS	Leadership indicator residual correlation freed	-2860.457	5816.913	6008.504	5856.197	.854	19	Normally identified; best LL replicated
CES WITH CMS	Colleague indicator residual correlation freed	-2869.662	5835.323	6026.914	5874.606	.822	26	Normally identified; best LL replicated
LES/LMS + CES/CMS	Leadership and colleague residual correlations freed	-2846.439	5790.878	5986.459	5830.979	.846	20	Normally identified; best LL replicated

Note. The model freeing OES WITH OMS generated a two-person profile and a non-positive definite information matrix and was therefore treated as a diagnostic model rather than an acceptable alternative. The joint LES/LMS + CES/CMS model was used as the principal local-dependence sensitivity model.

Supplementary Table S14. Joint local-dependence six-profile solution

Profile	Mplus class	n	%	OES	OMS	LES	LMS	CES	CMS
Very Low Overall Support Profile	1	20	5.00	-2.012	-1.798	-1.906	-1.788	-1.173	-1.116
Low Overall Support Profile	2	65	16.25	-.673	-.784	-.773	-1.193	-.462	-.795
Relational Support-Dominant Profile	3	27	6.75	-1.049	-1.121	.413	.382	.876	.953
Organizational Support-Dominant Profile	4	36	9.00	1.528	1.499	.620	.571	-.133	-.916
Moderate Balanced Support Profile	5	185	46.25	-.047	-.032	.048	.192	-.122	.075
High Balanced Support Profile	6	67	16.75	.981	1.035	.689	.729	.824	.998

Note. The joint model allowed LES WITH LMS and CES WITH CMS within profiles. Profile labels were aligned to the baseline solution by shape.

Supplementary Table S15. Classification agreement between the baseline and sensitivity models

Comparison	Statistic	Result
Five-indicator model vs. baseline	Person-level agreement	94.25%
Five-indicator model vs. baseline	Cohen's κ	.923
Five-indicator model vs. baseline	ARI	.852
Joint local-dependence model vs. baseline	Person-level agreement	89.00%
Joint local-dependence model vs. baseline	Cohen's κ	.851
Joint local-dependence model vs. baseline	ARI	.706

Note. Profile labels were aligned by profile shape before person-level agreement and Cohen's κ were computed. ARI is invariant to class-label permutations.

Supplementary Table S16. Cross-classification of the baseline and joint local-dependence six-profile solutions

Baseline profile	Joint: Very Low	Joint: Moderate	Joint: Low	Joint: Organizational	Joint: Relational	Joint: High	Row total
Very Low Overall Support Profile	20	0	1	0	0	0	21
Moderate Balanced Support Profile	0	152	4	0	3	2	161
Low Overall Support Profile	0	9	60	0	0	0	69
Organizational Support-Dominant Profile	0	0	0	36	0	1	37
Relational Support-Dominant Profile	0	8	0	0	24	0	32
High Balanced Support Profile	0	16	0	0	0	64	80

Note. Diagonal total = 356; N = 400; agreement = 89.00%.

6. Five-Indicator Sensitivity Analysis

Supplementary Table S17. Enumeration results for the five-indicator model

Profiles	LL	AIC	BIC	aBIC	Entropy	VLMR p	Smallest n	Smallest %
1	-2837.877	5695.754	5735.669	5703.938	—	—	—	—
2	-2654.703	5341.405	5405.269	5354.500	.742	.0417	132	33.00
3	-2541.682	5127.364	5215.176	5145.369	.852	.0284	61	15.25
4	-2500.532	5057.064	5168.825	5079.979	.838	.0755	42	10.50
5	-2443.664	4955.329	5091.038	4983.154	.868	.0031	31	7.75
6	-2414.769	4909.537	5069.196	4942.273	.846	.1249	18	4.50
7	-2396.762	4885.525	5069.132	4923.171	.867	.4432	7	1.75

Note. LS denotes the combined leadership-support indicator. The seven-profile solution produced a seven-person profile and only a negligible BIC improvement over the six-profile solution.

Supplementary Table S18. Five-indicator six-profile solution

Profile	Mplus class	n	%	OES	OMS	LS	CES	CMS
Very Low Overall Support Profile	1	18	4.50	-2.010	-1.805	-2.009	-1.214	-1.161
Moderate Balanced Support Profile	2	163	40.75	-.072	-.050	.111	-.283	-.056
Low Overall Support Profile	3	67	16.75	-.690	-.827	-1.077	-.508	-.808
Relational Support-Dominant Profile	4	32	8.00	-.866	-.877	.357	1.175	1.129
Organizational Support-Dominant Profile	5	37	9.25	1.519	1.501	.658	-.202	-.901
High Balanced Support Profile	6	83	20.75	.873	.908	.728	.920	1.040

Note. Indicators are standardized scores. Profile labels are based on sample-relative profile shapes.

Supplementary Table S19. Cross-classification of the baseline and five-indicator six-profile solutions

Baseline profile	Five-indicator: Very Low	Five-indicator: Moderate	Five-indicator: Low	Five-indicator: Organizational	Five-indicator: Relational	Five-indicator: High	Row total
Very Low Overall Support Profile	18	0	3	0	0	0	21
Moderate Balanced Support Profile	0	153	4	0	0	4	161
Low Overall Support Profile	0	9	60	0	0	0	69
Organizational Support-Dominant Profile	0	0	0	36	0	1	37
Relational Support-Dominant Profile	0	0	0	0	32	0	32
High Balanced Support Profile	0	1	0	1	0	78	80

Note. Diagonal total = 377; N = 400; agreement = 94.25%.

7. Distribution Across Anonymized Universities

Supplementary Table S20. Distribution of the baseline six profiles across anonymized universities

University	Very Low	Low	Moderate	Relational	Organizational	High	Total
University A	5 (4.6%)	13 (12.0%)	48 (44.4%)	8 (7.4%)	8 (7.4%)	26 (24.1%)	108
University B	5 (7.6%)	16 (24.2%)	24 (36.4%)	4 (6.1%)	8 (12.1%)	9 (13.6%)	66
University C	6 (5.4%)	20 (17.9%)	37 (33.0%)	13 (11.6%)	16 (14.3%)	20 (17.9%)	112
University D	2 (3.7%)	10 (18.5%)	24 (44.4%)	2 (3.7%)	4 (7.4%)	12 (22.2%)	54
University E	2 (4.2%)	8 (16.7%)	22 (45.8%)	5 (10.4%)	1 (2.1%)	10 (20.8%)	48
University F	1 (8.3%)	2 (16.7%)	6 (50.0%)	0 (0.0%)	0 (0.0%)	3 (25.0%)	12
Total	21 (5.25%)	69 (17.25%)	161 (40.25%)	32 (8.00%)	37 (9.25%)	80 (20.00%)	400

.Note. Values are n (within-university %), except for the Total row, which reports n (full-sample %). Pearson $\chi^2(25) = 23.604$, $p = .542$; Cramér's $V = .109$. Because several cells contain small counts, the chi-square result is interpreted descriptively. University names are anonymized as A–F.

Supplementary Table S21. Faculty Population and Final Sample Distribution Across the Six Participating Universities

University	Faculty population	Share of total faculty (%)	Final analytic sample (n)	Share of analytic sample (%)
University A	1614	26.90	108	27.00
University B	975	16.25	66	16.50
University C	1682	28.04	112	28.00
University D	808	13.47	54	13.50
University E	735	12.25	48	12.00
University F	185	3.08	12	3.00
Total	5999	100.00	400	100.00

Note. Universities are anonymized as A–F to preserve institutional confidentiality. Each university served as a sampling stratum, and sample allocations were determined approximately in proportion to each institution's share of the total faculty population across the six participating universities.

8. Questionnaire Adaptation and Measurement Documentation

Adaptation and scoring procedure. The questionnaire was administered in Chinese. The final battery was developed with reference to the original instruments, subsequent Chinese adaptations, and the questionnaire used by Xiong (2023), rather than through direct item-by-item retention from the full original scales. Items judged to be inapplicable to the teaching context of Chinese private universities were omitted during contextual adaptation. Support items were classified as emotional when they primarily reflected care, recognition, respect, encouragement, or psychological support, and as material when they primarily reflected tangible resources, information,

opportunities, task assistance, or practical support. Each dimension was scored as the mean of its retained items. No reverse-scored items were used in the final questionnaire. The final version underwent forward/back-translation, review by three academic experts, and pilot testing before formal administration.

Supplementary Table S22. Final Measurement Items and Dimension Assignment

Dimension	Item	Final administered item	Principal adaptation source(s)
GTE	B1	B1. University education plays a more important role in the growth and development of students than the influence of family and society.	Gibson & Dembo (1984); Yu et al. (1995); Xiong (2023)
		与家庭和社会的影响相比，大学教育在学生的成长与培养中发挥了更重要的作用。	
	B2	B2. Even if a student's motivation, perseverance, or ability to learn is lacking, a university education can still motivate them to make positive changes.	
		即使学生的学习动力、毅力或学习能力不足，大学教育依然能够促使他们产生积极的改变。	
	B3	B3.Students vary, but lecturers can help each student make progress based on their existing level of learning.	
		学生情况各异，大学教师能够帮助每位学生在其原有学习水平的基础上取得进步。	
	B4	B4. Among all the factors that affect students' academic achievement, the role of the lecturers is crucial.	
		在影响大学生学业成就的所有因素中，教师的作用是至关重要的。	
	B5	B5. Competent and enthusiastic lecturers are effective in helping students to improve their motivation, perseverance and ability to learn.	
		有能力且充满热情的教师能够有效帮助学生提升学习动力、毅力和学习能力。	
	B6	B6. Lecturers should not only teach students professional knowledge and skills, but should also play an important role in developing good character in students.	
		大学教师不仅应教授学生专业知识和技能，还应在培养学生良好品格方面发挥重要作用。	
PTE	B7	B7.Even in the face of unmotivated students in the classroom, I can take steps to help them improve their learning.	
		即使面对课堂上学习动力不足的学生，我仍然能够采取措施帮助他们改进学习状态。	
	B8	B8.If students forget the content of a previous lesson, I can help them recall and consolidate the knowledge through supplementary teaching.	
		如果学生忘记了之前课程的内容，我能够通过补充教学帮助他们回忆和巩固知识。	
	B9	B9.My teaching experience and practice has given me the skills and confidence necessary to meet the challenges of the classroom.	
		我的教学经历和实践让我获得了应对课堂挑战的必要技能和信心。	
	B10	B10.I can adjust the class schedule and teaching strategies according to the students' learning situation.	
		我能够根据学生的学习情况，调整课堂教学进度和教学策略。	
	B11	B11.If I encounter a new teaching task, I believe I can be competent and complete it.	
		如果遇到新的教学任务，我相信自己能够胜任并完成。	
	B12	B12.I believe that my teaching ability can handle all types of students, including those with low motivation or more complex backgrounds.	

Dimension	Item	Final administered item	Principal adaptation source(s)
		我相信自己的教学能力可以应对各种类型的学生，包括那些学习动力较低或背景较为复杂的学生。	
	B13	B13.I have the ability to support and mentor students who are struggling academically.	
		我有能力支持和指导那些学习困难的学生。	
	B14	B14.I can adjust my expectations to avoid putting too much pressure on some students due to their low starting points or limited learning conditions.	
		部分学生由于起点较低或学习条件有限，我能够调整自己的期望以避免对其施加过多压力。	
OES	C1	C1. The university takes my interests into account when making relevant teaching decisions.	Eisenberger et al. (1986); Chen & Liao (2006); Xiong (2023)
		学校做教学相关决策时会考虑我的利益。	
	C2	C2. The university encourages me to innovate in my classroom teaching in ways that reflect my own teaching style.	
		学校鼓励我根据自己的教学风格创新课堂教学。	
	C3	C3. The university cares about my physical and mental health and well-being (e.g. providing medical check-ups, psychological counselling services, etc.)	
		学校关心我的身心健康和幸福感（如提供体检、心理咨询服务等）。	
	C4	C4.The university's emphasis on the core status and value of lecturers has given me a sense of mission and achievement in my work as a lecturer.	
		学校重视教师的核心地位和价值，让我感受到作为教师工作的使命感和成就感。	
	C5	C5. University can notice and recognize lecturers who perform well and reward them or provide opportunities for career advancement to help them grow professionally.	
		学校会注意到并认可工作表现出色的教师，给予奖励或提供职业晋升的机会，帮助其实现职业成长。	
	C6	C6. The university pays attention to lecturers' teaching experience and establishes a feedback mechanism for lecturers.	
		学校关注教师的教学体验，并建立教师反馈机制。	
OMS	C7	C7. The university provides the necessary financial support for my teaching and research work.	
		学校为我的教学研究工作提供必要的配套资金支持。	
	C8	C8. The university provides me with fair and reasonable teaching assessment, professional title promotion, evaluation and other opportunities.	
		学校为我提供公平合理的教学考核、职称晋升、评优评先等机会。	
	C9	C9. The university provides me with timely and effective teaching management services.	
		学校为我提供了及时有效的教学管理服务。	
	C10	C10. The university provides me with abundant opportunities for teaching exchange, training, competition and further study.	
		学校为我提供了丰富的教学交流、培训、竞赛、进修等机会。	
	C11	C11. The university provides me with sufficient and high-quality teaching equipment and technological resources to support my classroom teaching.	
		学校为我提供了充足且高质量的教学设备和技术资源，以支持我的课堂教学。	

Dimension	Item	Final administered item	Principal adaptation source(s)
	C12	C12. The university supports me with a teaching development platform, a team and information materials.<br>学校为我提供了教学发展平台、团队和信息资料等支持。	
LES	D1	D1. My direct leader respects and supports my teaching philosophy and personal development goals, encouraging me to utilize my teaching expertise and enhance the quality of classroom instruction.<br>我的直属领导尊重并支持我的教学理念和个人发展目标，鼓励我发挥教学专长，提高课堂教学质量。	Kottke & Sharafinski (1988); Xiong (2023)
	D2	D2. When making decisions that affect my teaching work, my direct leader considers my needs and communicates with me sincerely.<br>当做出对我的教学工作有影响的决定时，我的领导会考虑到我的需求并真诚与我交流。	
	D3	D3. My direct leader cares about my teaching workload and overall well-being and provides necessary support.<br>我的直属领导关心我的教学工作压力和生活状态，并提供必要的帮助。	
	D4	D4. When I make unintentional mistakes in the teaching process, my direct supervisor provides understanding and support and helps me improve my teaching.<br>当我在教学过程中出现无心的错误时，我的直属领导会给予理解和支持，并帮助我改进教学。	
	D5	D5. If I do my best in teaching, my direct leader will undoubtedly notice my efforts and provide timely encouragement and feedback.<br>如果我尽力做好教学工作，我的直属领导一定会看到并及时给予鼓励和反馈。	
LMS	D6	D6. My direct leader assigns teaching tasks reasonably based on my teaching abilities and course characteristics to ensure effective teaching outcomes.<br>我的直属领导会根据我的教学能力和课程特点，合理安排教学任务，以确保教学效果。	
	D7	D7. When the teaching workload is heavy or involves curriculum reform, my direct leader coordinates teaching assistants or support staff to help me successfully complete my teaching tasks.<br>当教学工作量较大或涉及课程改革时，我的直属领导会协调助教或教学助手，帮助我顺利完成教学任务。	
	D8	D8. When I have a new teaching plan or idea, my direct leader provides the conditions or resources.<br>当我有新的教学计划或想法，我的直属领导会尽力提供条件或资源支持。	
	D9	D9. My direct leader encourages me to innovate in course instruction according to university regulations and grants me full academic autonomy.<br>我的直属领导根据学校规定鼓励我在教学中进行课程创新，并给予我充分的教学自主权。	
	D10	D10. My direct leader provides advice and opportunity on my teaching development planning and the improvement of my teaching abilities.<br>我的直属领导会为对我的教学发展规划和教学能力提升提供建议和机会。	
CES	E1	E1. Colleagues respect my opinion on work that requires mutual co-operation.<br>在需要相互配合的工作上，同事尊重我的意见。	Ladd & Henry (2000); Xiong (2023)
	E2	E2. In the face of teaching pressure, colleagues can give me psychological encouragement and support.<br>在面对教学压力时，同事会给予我心理上的鼓励和支持。	

Dimension	Item	Final administered item	Principal adaptation source(s)
	E3	E3.My colleagues can communicate with me effectively and clarify relevant teaching requirements.	
		同事能与我有效沟通、明确相关教学工作要求。	
	E4	E4. I receive congratulations and encouragement from colleagues when I make progress or achievements in teaching.	
		在教学中取得进步或成绩时，我会收到同事的祝贺和鼓励。	
	E5	E5. My colleagues create a positive, good and friendly teaching atmosphere.	
		我的同事会营造出积极、良好、友善的教学氛围。	
CMS	E6	E6. If there are channels, my colleagues are willing to provide the relevant information and materials required by the work for me(lesson plans, courseware, teaching cases, etc.).	
		如果有渠道，同事愿意为我提供工作所需的相关信息和资料（教案、课件、教学案例等）。	
	E7	E7. When I encounter technical problems with teaching, my colleagues offer advice or help to solve them.	
		当我遇到教学技术问题时，同事会提供建议或帮忙解决。	
	E8	E8. My colleagues will cooperate with me to complete the teaching work.	
		同事会与我默契配合完成教学工作。	
	E9	E9. During the process of awards selection, performance evaluation, promotion, or further study applications, my colleagues provide formal recommendations or assistance to ensure that I receive fair opportunities.	
		在评优评先、考核晋升或进修申请过程中，同事会提供正式推荐或协助，确保我获得公平的机会。	
	E10	E10. If I can't finish the relevant teaching work because of emergency, I will get help from my colleagues.	
		如果有急事不能完成相关教学工作，我会得到同事的帮助。	

9. Additional Construct Validity and Discriminant Validity Evidence

Supplementary Table S23. Latent-Factor Correlations Among the Eight Constructs

Construct	GTE	PTE	OES	OMS	LES	LMS	CES	CMS
GTE	—							
PTE	0.839	—						
OES	0.500	0.497	—					
OMS	0.543	0.535	0.838	—				

LES	0.523	0.411	0.586	0.544	—			
LMS	0.455	0.398	0.626	0.597	0.886	—		
CES	0.394	0.428	0.305	0.327	0.534	0.506	—	
CMS	0.257	0.310	0.217	0.252	0.506	0.529	0.778	—

Note. N = 400. Values are standardized latent-factor correlations obtained from the eight-factor WLSMV confirmatory factor analysis. GTE = General Teaching Efficacy; PTE = Personal Teaching Efficacy; OES = Organizational Emotional Support; OMS = Organizational Material Support; LES = Leadership Emotional Support; LMS = Leadership Material Support; CES = Colleague Emotional Support; CMS = Colleague Material Support.

Supplementary Table S24. Heterotrait-Monotrait Ratio (HTMT) Matrix for the Eight Constructs

Construct	GTE	PTE	OES	OMS	LES	LMS	CES	CMS
GTE	—							
PTE	0. 834	—						
OES	0. 49	0. 486	—					
OMS	0. 527	0. 526	0. 828	—				
LES	0. 52	0. 401	0. 587	0. 532	—			
LMS	0. 445	0. 392	0. 624	0. 586	0. 872	—		
CES	0. 367	0. 401	0. 292	0. 306	0. 491	0. 477	—	
CMS	0. 252	0. 312	0. 215	0. 256	0. 487	0. 519	0. 743	—

Note. N = 400. HTMT values were calculated from the retained item-level responses for the eight constructs. The LES–LMS HTMT value (.872) was the highest among the construct pairs, indicating comparatively substantial overlap between the two leadership-support dimensions. GTE = General Teaching Efficacy; PTE = Personal Teaching Efficacy; OES = Organizational Emotional Support; OMS = Organizational Material Support; LES = Leadership Emotional Support; LMS = Leadership Material Support; CES = Colleague Emotional Support; CMS = Colleague Material Support.

10. Variance-Specification Sensitivity Analysis

Supplementary Table S25. Comparison of Equal- and Profile-Specific Variance Models

Variance specification	Profiles	Free parameters	LL	AIC	BIC	aBIC	Entropy	Smallest n	Smallest %

Equal within-profile variances; covariances fixed at 0	6	47	-2889.984	5873.968	6061.567	5912.433	0.850	21	5.25%
Profile-specific variances; covariances fixed at 0	5	64	-2803.513	5735.027	5990.480	5787.404	0.859	32	8.00%
Profile-specific variances; covariances fixed at 0	6	77	-2759.941	5673.882	5981.225	5736.899	0.854	31	7.75%

Note. LL = loglikelihood; AIC = Akaike information criterion; BIC = Bayesian information criterion; aBIC = sample-size-adjusted BIC. All models used the same six standardized support indicators and fixed within-profile covariances at zero. The focal five-versus-six comparison under the profile-specific-variance specification favored six profiles on AIC, BIC, and aBIC.

Supplementary Table S26. Six-Profile Solution Under Profile-Specific Variances

Profile	Mplus class	n	%	OES	OMS	LES	LMS	CES	CMS
Low Overall Support Profile	1	56	14.00%	-1.044	-0.959	-1.323	-1.523	-0.673	-0.947
Below-Average Balanced Support Profile	2	106	26.50%	-0.443	-0.480	-0.408	-0.368	-0.366	-0.404
Moderate Balanced Support Profile	3	138	34.50%	0.176	0.179	0.275	0.377	0.001	0.242
Relational Support-Dominant Profile	4	32	8.00%	-0.492	-0.423	0.861	0.620	1.398	1.420
Organizational Support-Dominant Profile	5	31	7.75%	1.658	1.573	0.853	0.730	-0.114	-0.790
High Balanced Support Profile	6	37	9.25%	1.261	1.232	0.775	0.905	1.065	1.262

Note. Indicators are full-sample z-standardized scores. Within-profile variances were allowed to differ across profiles, while within-profile covariances were fixed at zero. Profile labels are descriptive and based on the relative shapes of the estimated means.

Supplementary Table S27. Cross-Classification of Baseline and Profile-Specific-Variance Six-Profile Solutions

Baseline profile	Unequal: Low Overall	Unequal: Below-Average Balanced	Unequal: Moderate Balanced	Unequal: Relational	Unequal: Organizational	Unequal: High Balanced	Row total
Very Low Overall Support Profile	**21**	0	0	0	0	0	21
Low Overall Support Profile	24	**45**	0	0	0	0	69
Moderate Balanced Support Profile	3	58	**100**	0	0	0	161
Relational Support-Dominant Profile	5	3	4	**20**	0	0	32
Organizational Support-Dominant Profile	3	0	4	0	**30**	0	37
High Balanced Support Profile	0	0	30	12	1	**37**	80

Column total	56	106	138	32	31	37	400
**Aligned person-level agreement**	63.25%						
**Cohen's κ**	0.526						
**Adjusted Rand index**	0.373						

Note. Rows represent the baseline equal-variance six-profile solution; columns represent the six-profile solution with profile-specific indicator variances. The highlighted cells define the profile alignment used to calculate person-level agreement. Exact profile membership changed materially across variance specifications, although the broader support-level gradient and the two source-dominant configurations remained recognizable.

11. Scaling Sensitivity Analysis

Supplementary Table S28. Enumeration Results for the LPA Using Original 1–5 Scale Scores

No. of profiles	Free parameters	LL	AIC	BIC	aBIC	Entropy	VLMR p	Smallest n	Smallest %
1	12	-2166.032	4356.064	4403.962	4365.885			400	100.00%
2	19	-1899.929	3837.858	3913.695	3853.407	0.826	< .001	114	28.50%
3	26	-1788.558	3629.116	3732.894	3650.395	0.838	.011	62	15.50%
4	33	-1733.876	3533.752	3665.470	3560.759	0.793	.146	44	11.00%
5	40	-1688.990	3457.980	3617.638	3490.716	0.862	.187	31	7.75%
**6**	**47**	**-1650.564**	**3395.128**	**3582.726**	**3433.592**	**0.850**	**.131**	**21**	**5.25%**
7	54	-1628.150	3364.301	3579.840	3408.494	0.845	.098	2	0.50%

Note. LL = loglikelihood; AIC = Akaike information criterion; BIC = Bayesian information criterion; aBIC = sample-size-adjusted BIC; VLMR = Vuong–Lo–Mendell–Rubin likelihood-ratio test. The models were estimated using the six support-dimension means on their original 1–5 metric. The seven-profile solution produced a two-person profile and a non-positive definite first-order derivative product matrix, indicating possible nonidentification. Bold values indicate the retained six-profile representation.

Supplementary Table S29. Six-Profile Means on the Original 1–5 Scale

Profile	Mplus class	n	%	Posterior probability	OES	OMS	LES	LMS	CES	CMS
Very Low Overall Support Profile	1	21	5.25%	0.919	2.514	2.737	2.962	2.312	3.719	3.124
Low Overall Support Profile	3	69	17.25%	0.865	3.354	3.435	3.527	2.834	3.975	3.354

Profile	Mplus class	n	%	Posterior probability	OES	OMS	LES	LMS	CES	CMS
Moderate Balanced Support Profile	5	161	40.25%	0.893	3.728	3.871	4.015	3.876	4.062	3.836
Relational Support-Dominant Profile	4	32	8.00%	0.892	3.199	3.315	4.221	3.959	4.599	4.578
Organizational Support-Dominant Profile	2	37	9.25%	0.931	4.754	4.914	4.340	4.191	4.087	3.285
High Balanced Support Profile	6	80	20.00%	0.895	4.328	4.511	4.365	4.306	4.507	4.539
**Full-sample reference**					3.770	3.915	3.992	3.735	4.165	3.867
**Full-sample SD**					0.648	0.667	0.542	0.785	0.381	0.648

Note. Values for OES, OMS, LES, LMS, CES, and CMS are estimated profile-specific means on the original 1–5 metric. Profile labels and ordering correspond to the primary z-standardized six-profile solution. Posterior probability refers to the average latent-class probability for cases assigned to the corresponding most-likely class. The full-sample reference rows are included to show the original-score location and dispersion of each support indicator.

Supplementary Table S30. Intraclass Correlations for Study Variables Across Universities

Variable	ICC
GTE	0.011
PTE	0.033
OES	< .001
OMS	< .001
LES	0.013
LMS	< .001
CES	< .001
CMS	0.019

Note. ICCs were estimated from unconditional random-intercept models with university as the grouping variable (N = 400; six universities). ICC = between-university variance / (between-university variance + within-university variance). Values below .001 are reported as < .001. Given the small number of universities, the ICCs are intended as descriptive indicators of institutional clustering.

12. Correlation Structure and Distributional Information

Supplementary Table S31. Observed-Score Correlations and Distributional Statistics

Panel A. Pearson correlations among the eight study variables								
**Variable**	**OES**	**OMS**	**LES**	**LMS**	**CES**	**CMS**	**GTE**	**PTE**
**OES**	—							

**OMS**	0.752	—						
**LES**	0.483	0.444	—					
**LMS**	0.515	0.490	0.661	—				
**CES**	0.230	0.245	0.354	0.346	—			
**CMS**	0.183	0.220	0.385	0.408	0.556	—		
**GTE**	0.420	0.456	0.409	0.352	0.274	0.202	—	
**PTE**	0.402	0.438	0.304	0.296	0.290	0.238	0.652	—

**Panel B. Distributional statistics for the six LPA indicators**		
**Variable**	**Skewness**	**Excess kurtosis**
**OES**	-0.077	-0.237
**OMS**	-0.130	-0.493
**LES**	-0.452	-0.314
**LMS**	-0.838	0.181
**CES**	0.043	0.520
**CMS**	-0.251	-0.194

Note. N = 400. Panel A reports Pearson correlations based on observed scale scores. All reported correlations were statistically significant at p < .001. Panel B reports skewness and excess kurtosis for the six support indicators used in the latent profile analysis. GTE = General Teaching Efficacy; PTE = Personal Teaching Efficacy; OES = Organizational Emotional Support; OMS = Organizational Material Support; LES = Leadership Emotional Support; LMS = Leadership Material Support; CES = Colleague Emotional Support; CMS = Colleague Material Support.

13. Mplus Model Specifications and Reproducibility

Supplementary Table S32. Summary of Mplus Specifications for the Primary and Sensitivity Analyses

Analysis	Indicators / variables	No. of profiles	Scaling	Within-profile variances	Within-profile covariances	Estimator / procedure	STARTS	STITER ATIONS	Key specification / purpose
Primary six-profile LPA	OES, OMS, LES, LMS, CES, CMS	6	Full-sample z scores	Constrained equal across profiles	Fixed at 0	MLR mixture model	16000 3200	20	Primary model used for substantive interpretation; profile-specific means freely estimated.
Five-profile competing solution	OES, OMS, LES, LMS, CES, CMS	5	Full-sample z scores	Constrained equal across profiles	Fixed at 0	MLR mixture model	10000 2000	20	More parsimonious competing representation used to assess whether the lower end of the support continuum required further subdivision.

Local-dependence sensitivity model	OES, OMS, LES, LMS, CES, CMS	6	Full-sample z scores	Constrained equal across profiles	LES WITH LMS; CES WITH CMS	MLR mixture model	25000 5000	20	Tests sensitivity to selected within-source residual dependence while retaining the six-profile structure.
Broader covariance diagnostic model	OES, OMS, LES, LMS, CES, CMS	6	Full-sample z scores	Constrained equal across profiles	OES WITH OMS; LES WITH LMS; CES WITH CMS	MLR mixture model	20000 4000	20	Diagnostic model for a broader covariance specification; estimation instability prevented substantive interpretation.
Combined-leadership sensitivity model	OES, OMS, LS, CES, CMS; LS = (LES + LMS) / 2	6	Full-sample z scores	Constrained equal across profiles	Fixed at 0	MLR mixture model	10000 2000	20	Evaluates whether the primary profile structure depends on modeling LES and LMS as separate indicators.
Profile-specific variance sensitivity model	OES, OMS, LES, LMS, CES, CMS	6	Full-sample z scores	Freely estimated across profiles	Fixed at 0	MLR mixture model	30000 6000	20	Evaluates sensitivity of profile number, shapes, and classification to the equal-variance assumption.
Raw-score scaling sensitivity model	OES, OMS, LES, LMS, CES, CMS	6	Original 1–5 dimension means	Constrained equal across profiles	Fixed at 0	MLR mixture model	16000 3200	20	Re-estimates the selected model without z-standardization to assess scaling sensitivity.
BCH weight-generation step	Six support indicators; GTE and PTE retained as auxiliary/distal variables	6	Primary LPA specificati on	As in primary six-profile model	As in primary six-profile model	Manual BCH / weight generation	—	—	Reproduces the selected six-profile solution and saves BCH weights; GTE and PTE do not contribute to profile formation.
BCH distal-outcome model: GTE	GTE as distal outcome + saved BCH weights	6	Outcome on original 1–5 metric	Not applicable	Not applicable	BCH distal-outcome comparison	—	—	Compares BCH-adjusted GTE means across the selected profiles; pairwise p values corrected using Holm procedure.
BCH distal-outcome model: PTE	PTE as distal outcome + saved BCH weights	6	Outcome on original 1–5 metric	Not applicable	Not applicable	BCH distal-outcome comparison	—	—	Compares BCH-adjusted PTE means across the selected profiles; pairwise p values corrected using Holm procedure.

Note. OES = Organizational Emotional Support; OMS = Organizational Material Support; LES = Leadership Emotional Support; LMS = Leadership Material Support; CES = Colleague Emotional Support; CMS = Colleague Material Support; LS = combined leadership support. MLR = robust maximum likelihood. A dash indicates that the setting was not separately reported in the retained analysis record or was not applicable to the BCH stage. Alternative models retained the same general mixture-model framework unless otherwise noted.

Representative Mplus Syntax for the Primary Model

```
VARIABLE:
  NAMES = id oes oms les lms ces cms;
  USEVARIABLES = oes oms les lms ces cms;
  IDVARIABLE = id;
```

```
  CLASSES = c(6);

DEFINE:
  STANDARDIZE oes oms les lms ces cms;

ANALYSIS:
  TYPE = MIXTURE;
  ESTIMATOR = MLR;
  STARTS = 16000 3200;
  STITERATIONS = 20;

OUTPUT:
  SAMPSTAT TECH1 TECH4 TECH8 TECH11;

SAVEDATA:
  SAVE = CPROBABILITIES;
```

The alternative models used the same basic syntax, with the modifications summarized in Supplementary Table S32.

14. Verification Summary

The eight-factor measurement model showed good overall fit. Merging GTE/PTE, OES/OMS, or CES/CMS significantly worsened fit; merging LES/LMS did not significantly worsen fit, consistent with empirical overlap between the two leadership-support dimensions.

The six-profile solution was stable under extensive random starts and produced interpretable profiles with acceptable posterior classification probabilities. The seven-profile solution generated a two-person profile and an identification warning.

BCH analyses showed significant overall differences in GTE and PTE. Holm-corrected comparisons supported especially low efficacy in the Very Low Overall Support Profile and high efficacy in the Organizational Support-Dominant and High Balanced profiles. Nonsignificant differences were not interpreted as evidence of statistical equivalence.

The five-profile competing solution reproduced four major profile shapes and represented the lower end of the support continuum as one broad Low Overall Support Profile, supporting the interpretation that the six-profile solution further differentiated low-support teachers by severity.

The joint local-dependence model and the five-indicator model reproduced the principal profile shapes and showed high classification agreement with the baseline solution. Models freeing OES WITH OMS produced an inadmissibly small profile and were treated as diagnostic only.

The six-profile distribution did not differ significantly across the six anonymized universities.
